# Two new species of Cyatholaimidae (Nematoda: Chromadorida) from the Southeastern Brazilian coast with emphasis on the pore complex and lateral pore-like structures

**DOI:** 10.7717/peerj.14712

**Published:** 2023-02-20

**Authors:** Beatriz P. Cunha, Gustavo Fonseca, Antonia Cecília Z. Amaral

**Affiliations:** 1 Departamento de Biologia Animal, Instituto de Biologia, Universidade Estadual de Campinas, Campinas, São Paulo, Brazil; 2 Instituto do Mar, Universidade Federal de São Paulo, Santos, São Paulo, Brazil

**Keywords:** Meiofauna, Taxonomy, Marine nematodes, Tabular key, SEM, rDNA SSU

## Abstract

Cyatholaimidae is a common and diverse family of mainly marine nematodes, potentially, with a large number of species to be discovered. The taxonomy of the group is marked by a lack of information about the evolutionary history of the characters and of detailed descriptions of morphological structures that may be taxonomically relevant. Two new species of the family are described from a sublittoral region in Southeastern Brazil, emphasizing the importance of the distribution and morphology of pore complex and pore-like structures present on the cuticle. The taxonomic importance of the cuticle ornamentation and spicule shape for the *Biarmifer* species, as well as the precloacal supplements structures of *Pomponema* species, are discussed. *Biarmifer nesiotes* sp. nov. differs from other species of the genus by the presence of eight longitudinal rows of pore complex on the cuticle and by the shape of the copulatory structure. *Pomponema longispiculum* sp. nov. differs from the most similar species, *P. stomachor* Wieser, 1954, by the smaller number of turns of the amphidial fovea, the shorter tail and the beginning of the cuticle lateral differentiation (3/4 of the pharynx length *vs*. end of the pharynx, respectively). We also obtained the SSU rDNA sequence from *Pomponema longispiculum* sp. nov., which is closely related to *Pomponema* sp. (MN250093) by about 91%. Updated tabular keys to species identification of each genus (*Biarmifer* and *Pomponema*) are included, containing morphometric data, characters related to cuticle ornamentation, and copulatory structures.

## Introduction

Free-living marine nematodes are one of the most abundant and diverse groups of the meiofauna, however, the lack of phylogenetically informative morphological characters can hamper the study of relationships between and within taxa (Pereira et al., 2010). A careful observation of morphological characters through optical and electron microscopy combined with molecular data acquisition expands our knowledge about marine nematode taxonomy and allows the understanding of the evolution of the shape and form of species (Fonseca, Fontaneto & Di Domenico, 2017).

The family Cyatholaimidae Filipjev, 1918 is a diverse group of mainly marine nematodes with 20 genera and 211 valid species distributed worldwide in different types of habitats (Cunha, Fonseca & Amaral, 2022). Based on morphology, it has been subdivided into four subfamilies (Cyatholaiminae Filipjev, 1918, Paracanthonchinae De Coninck, 1965, Pomponematinae Gerlach & Riemann, 1973 and Xenocyatholaiminae Gerlach & Riemann, 1973), however, none are supported by any synapomorphy (Lorenzen, 1981). The same is true for many genera of the family, which are delimited by characters that may not be phylogenetically relevant (Cunha, Fonseca & Amaral, 2022). Therefore, taxonomic studies of Cyatholaimidae are marked by changes in classifications, synonyms and transfers of species between genera (*e.g*., Micoletzky, 1924; Wieser, 1954; Miljutina & Miljutin, 2015).

For instance, the genus *Pomponema* was first erected by Cobb (1917) and later reviewed by Lorenzen (1972), who provided a diagnosis of the genus, based on the complex precloacal supplements, the well-developed buccal cavity and cuticle differentiation not like in *Craspodema* Gerlach, 1956 (lateral differentiation in the pharynx region as longitudinal rows of enlarged punctations with broadly lateral fields between them; at the end of the pharynx, these punctations became smaller and eight additional longitudinal fields became evident throughout the body). The author considered the genera *Anaxonchium* Cobb, 1920, *Nummocephalus* Filipjev, 1946, *Haustrifera* Wieser, 1954, and part of the genera *Cyatholaimus* Bastian, 1865 and *Longicyatholaimus* Micoletzky, 1924 as synonyms of *Pomponema*. Recently, *Parapomponema* Ott, 1972 and *Propomponema* Ott, 1972 were synonymized with this genus (Cidreira et al., 2019). Unlike *Pomponema*, the genus *Biarmifer* Wieser, 1954 underwent fewer changes. The genus was erected by Wieser (1954) who described two species and, in 1959, the author erected the new species *Biarmifer gibber*, which was synonymized with *Paracanthonchus longus* Allgén, 1934 by Lorenzen (1972). The genus was only found again over 40 years later when Pastor de Ward (2001) described *Biarmifer madrynensis*. Based on the cuticle ornamentation, which is unique to this group, it was recently proposed the reclassification of three species belonging to *Marylynnia* (Hopper, 1972) Hopper, 1977 to *Biarmifer* (Cunha, Fonseca & Amaral, 2022).

Pore complexes and lateral pore-like structures (LP) are cuticle structures with unknown functions commonly observed in cyatholaimids (Leduc & Zhao, 2016). Despite being poorly known, the size, structure, and cuticularization of both structures seem to diverge among species and may be taxonomically relevant (Hopper, 1972; Leduc & Zhao, 2016). The pore complexes are ring-like structures of dense material in the middle cuticle layer and are commonly seen along the sublateral, subventral, and subdorsal lines. They are characterized as a channel with a slit-like aperture and an associated cell (Wright & Hope, 1968; Leduc & Zhao, 2016). These pores vary in number, size, and organization, being distributed irregularly along the body or in four to twelve longitudinal rows (Hopper, 1972). The lateral pore-like is rarely included in species description, mostly due to its inconspicuous nature, hardly seen in detail on light microscopy. They are usually observed along the mediolateral lines of the body and are composed of a circular cuticularized opening supported by punctations and with a central dome (Leduc & Zhao, 2016).

The number of marine nematode species recorded in Brazil is low considering its long and heterogeneous coastline of almost 7,500 km. For the Cyatholaimidae family, only 20 species belonging to 13 genera have been registered (Venekey, Fonseca-Genevois & Santos, 2010; Venekey, 2017; Oliveira et al., 2017; Cidreira et al., 2019). However, some local surveys on different habitats of the country found a high diversity of the family. In the intertidal zone of a beach in the state of Rio de Janeiro, for example, six genera of the family were identified, being the most diverse taxa along with Chromadoridae (Maria et al., 2013). More than ten morphospecies of Cyatholaimidae were found in Araçá Bay, a tidal flat located in the state of São Paulo (Vieira & Fonseca, 2019). These findings, together with the low number of studies, suggest that there are many species to be discovered.

In the present study, we describe two new Cyatholaimidae species from a subtidal region of a beach on the Southeast Brazilian coast using morphological and molecular (SSU rDNA) data. We also analyzed the distribution and morphology of the pore complex and pore-like structures using light and scanning electron microscopy (SEM) and discuss its variabilities on species from the family.

## Materials & Methods

### Sampling and morphological data

**Figure 1:**
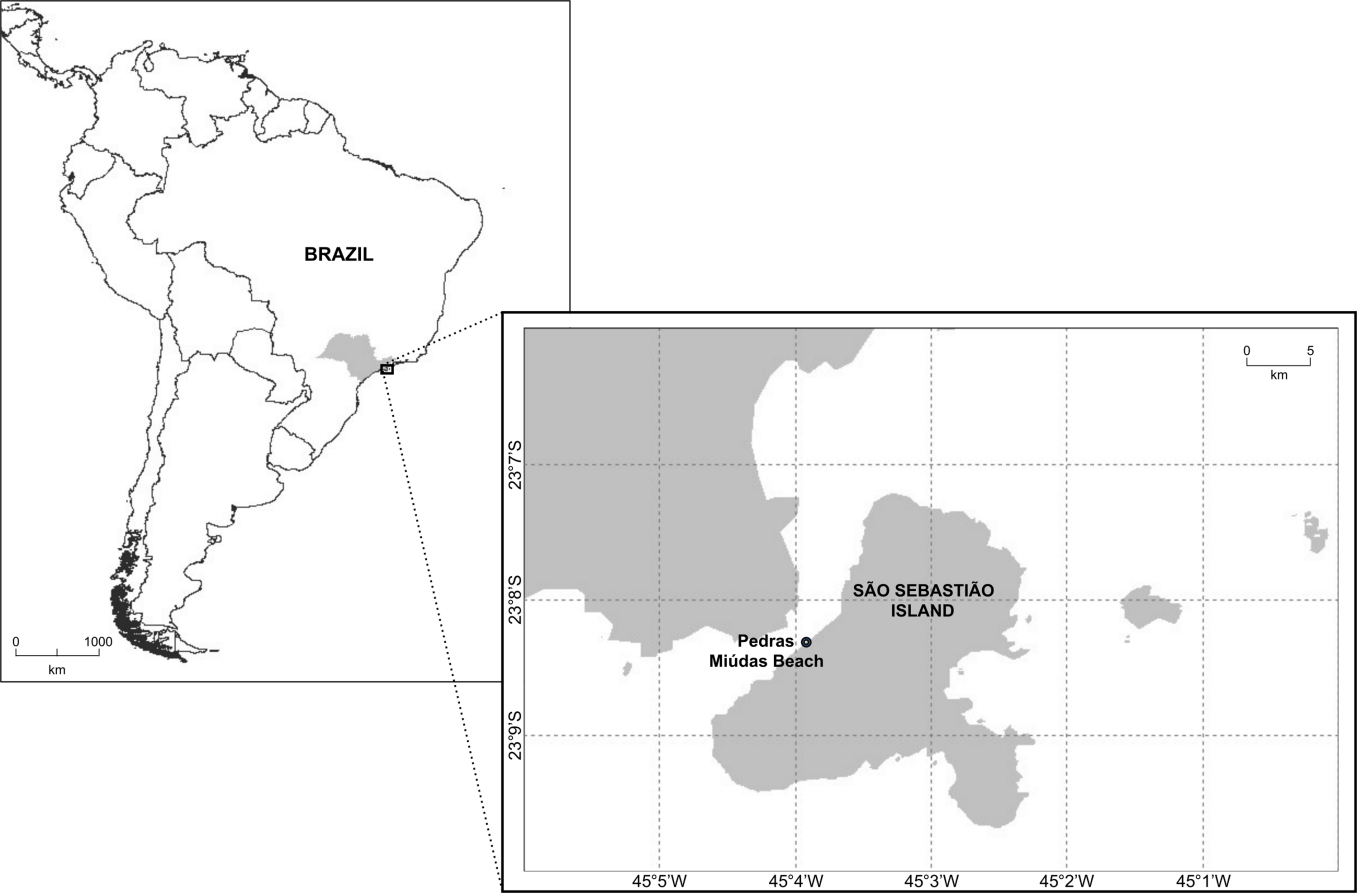
Map showing the location of Pedras Miúdas beach at São Sebastião Island, São Paulo, Brazil.

Sampling was conducted in February 2020, in the subtidal zone of Pedras Miúdas Beach, which is less than 80 m long (Fig. 1). This beach is located at São Sebastião Island, the main island of the Ilhabela Archipelago, with 337 km^2^. It is separated from the mainland by the São Sebastião Channel, which in its narrowest part is approximately 1.8 km wide. The collection was approved by ICMBio (19887-1).

The specimens were extracted from qualitative samples from the top five cm of sediment (1–2 m water depth). The sediment was frozen at −20 °C and, in the laboratory, it was thawed overnight before the fauna extraction using Ludox TM-50 with a density of 1.18 g cm^−3^ (Heip, Vincx & Vranken, 1985). The nematode species were sorted using a stereomicroscope. Part of the specimens was fixed in DESS (Yoder et al., 2006), transferred to glycerin and mounted onto permanent slides for light microscopy under a ZEISS Imager.M2 microscope equipped with differential interference contrast (DIC). The measurements were performed using images taken by an AxioCam MRc5 camera and its software. All measurements are in micrometers (µm) and all curved structures are measured along the arc. In addition to standard body measurements, we provide information on the number of mediolateral pore-like structures and sublateral pore complexes in the pharyngeal, tail, and central body regions (between the end of the pharynx and anal region) following Hopper (1972). Those numbers were determined for the uppermost and the most visible row. Abbreviations of measurements are as follows: a, body length/maximum body diameter; abd, anal body diameter; b, body length/pharynx length; c, body length/tail length; c′, tail length/anal or cloacal body diameter; cbd, corresponding body diameter; L, total body length; LP, lateral pore-like structures; PC, pore complexes; V, vulva distance from anterior end of body; %V, position of vulva as a percentage of body length from anterior end.

At least one adult individual of each species studied was fixed in 2.5% glutaraldehyde solution with sodium cacodylate buffer for SEM observation. Specimens were then dehydrated in a graded ethanol series (25% during 30 min and 30, 50, 70, 80, 90, 95, first 100, and second 100% changing from one concentration to the following every 10 min) and submitted to critical point drying. Next, individuals were mounted onto stubs and coated with gold/palladium using a sputter coater. Observations were made using a JSM 5800LV tabletop scanning electron microscope at high vacuum mode at the Electron Microscope Laboratory of the Institute of Biology at the University of Campinas (LME/UNICAMP). Holotypes and paratypes were deposited on MDBio—Museum of Biological Diversity of the Institute of Biology at the University of Campinas, Brazil (ZUEC-NMA/Collection).

The information about measurements and character states presented in the tabular keys were retrieved from the original descriptions. If a character was not reported in the text of a description, it was measured/described based on the associated illustrations.

The electronic version of this article in Portable Document Format (PDF) will represent a published work according to the International Commission on Zoological Nomenclature (ICZN), and hence the new names contained in the electronic version are effectively published under that Code from the electronic edition alone. This published work and the nomenclatural acts it contains have been registered in ZooBank, the online registration system for the ICZN. The ZooBank LSIDs (Life Science Identifiers) can be resolved and the associated information viewed through any standard web browser by appending the LSID to the prefix http://zoobank.org/. The LSID for this publication is: urn:lsid:zoobank.org:pub:2430E61E-EDC6-410B-B7CE-B24C1AA6CA0B. The online version of this work is archived and available from the following digital repositories: PeerJ, PubMed Central SCIE and CLOCKSS.

### Molecular data

The small ribosomal subunit (18S) was selected since it has been traditionally used in taxonomy and metabarcoding studies of marine nematodes (*e.g.*, Derycke et al., 2010; Armenteros et al., 2014). Previous to molecular analysis, one adult individual of each species was mounted in a drop of water on temporary slides to be photographed. The DNA extraction of each specimen was done with Worm lysis buffer following Derycke et al. (2005).

Primers for the rDNA SSU were forward primer 18S-CL-F, 5′-TCAAAGATTAAGCCATGCAT-3′ (Carta & Li, 2018) and reverse primer 1912BR, 5′-TTTACGGTTAGAACTAGGG-3′ (adapted from Holterman et al., 2006). The PCR reaction was performed in total volumes of 20 µl containing 10 µl Go Taq® Green Master Mix (Promega Corporation, Madison, WI, USA), 1 µl (10 µM) each of forward and reverse primers, and 2 to 5 µl of DNA template, depending on the DNA concentration. Cycling conditions for the amplification were: denaturation at 94 °C for 5 min, followed by 35 cycles of denaturation at 94 °C for 30 s, annealing at 50 °C for 30 s, and extension at 72 °C for 2 min, and a final extension at 72 °C for 10 min. The amplified products were sequenced using a Sanger ABI 3500xL sequencer at the Multi-User Genotyping and Sequencing Laboratory of the Institute of Biology at the State University of Campinas. The sequence was observed and edited in the Geneious Premium 2022.1 program (http://www.geneious.com). Unfortunately, the amplification of DNA for the *Biarmifer* species was not successful. The DNA sequence of the *Pomponema* species was deposited in the GenBank under the accession number OP548510. The light micrographics of this *Pomponema* individual used for molecular analyses was deposited on MDBio (Museum of Biological Diversity of the Institute of Biology at the University of Campinas) under the accession number ZUEC-PIC 700 (available at https://www2.ib.unicamp.br/fnjv/).

## Results

### Taxonomy

**Table:** 

Class Chromadorea Inglis, 1983
Order Chromadorida Chitwood, 1933
Family CYATHOLAIMIDAE Filipjev, 1918

**Diagnosis: (from Leduc & Zhao, 2016)** Cuticle with transverse rows of punctations. Lateral punctations may be larger, irregular or arranged in longitudinal rows. Longitudinal rows of circular or elliptical cuticular structures (termed ‘lateral pore-like structures’) often present along mediolateral lines; up to 12 longitudinal rows of pore complexes may also be present. Inner labial sensilla often setiform; six outer labial setae and four cephalic setae in a single crown (with some rare exceptions); outer labial setae longer than the cephalic setae. Multispiral amphidial fovea. Cheilostoma with twelve distinctly cuticularized rugae. Pharyngostoma with a large dorsal tooth, and usually with two smaller ventrosublateral teeth, which may be single or double. Pharynx usually without a posterior bulb. Female didelphic-amphidelphic with reflexed anterior and posterior gonads always on opposite sides of the intestine. Male usually with two testes, rarely with one. Precloacal supplements may be present or absent.

**Table:** 

Subfamily CYATHOLAIMINAE Filipjev, 1918

**Diagnosis: (emended from Decraemer & Smol, 2006)** Body cuticle with homogeneous punctation (except in *Biarmifer*), with or without lateral differentiation. Precloacal supplements absent or cup-shaped (tubular in *Praeacanthonchus*); gubernaculum unpaired proximally, except in *Biarmifer* and *Marylynnia* (paired).

**Table:** 

Genus *Biarmifer*Wieser, 1954

**Diagnosis: (emended from Pastor de Ward, 2001)** Cuticle heterogeneous, with enlarged hexagonal punctuations in transverse rows from the anterior end to the nerve ring and transverse rows of simple punctuations on the rest of the body. Lateral differentiation absent or of more widely spaced punctations; anterior punctuations without stellate processes. Pore complexes arranged in four or eight longitudinal rows, with slit-like aperture variable, at 0–90° angles to the longitudinal body axis. One median-sized dorsal tooth and two pairs of small sub-ventral teeth present. Spicules with inner processes and simple distal end, gubernaculum distally expanded or not. Cup-shaped non-sclerotized precloacal supplementary organs. Tail conical with or without a filiform portion.

**Remarks:** the genus *Biarmifer* was considered as being part of the Paracanthonchinae subfamily by Gerlach & Riemann (1973). A revaluation of the literature led Pastor de Ward (2001) to transfer the genus to the subfamily Cyatholaiminae mostly due to the cup-shaped precloacal supplements (it is tubular in Paracanthonchinae). The cuticle ornamentation of enlarged hexagonal punctations in the anterior end is a unique characteristic of the genus. Based on that, the species *Marylynnia dayi* (Inglis, 1963) Hopper, 1972, *M. hopperi* Sharma & Vincx, 1982, and *M. punctata* Jensen, 1985 were transferred to *Biarmifer* by Cunha, Fonseca & Amaral (2022). Besides cuticle ornamentation, both genera may be differentiated by the number of longitudinal rows of pore complexes. *Marylynnia* species have up to 12 rows (with a few exceptions, like *M. oculissoma* (Hopper, 1972) Hopper, 1977), and *Biarmifer* presents four longitudinal rows. Of the three *Marylynnia* species transferred, only *M. hopperi* has eight rows of pore complexes, the other two species possess four rows.

**Table:** 

**Figure 2:**
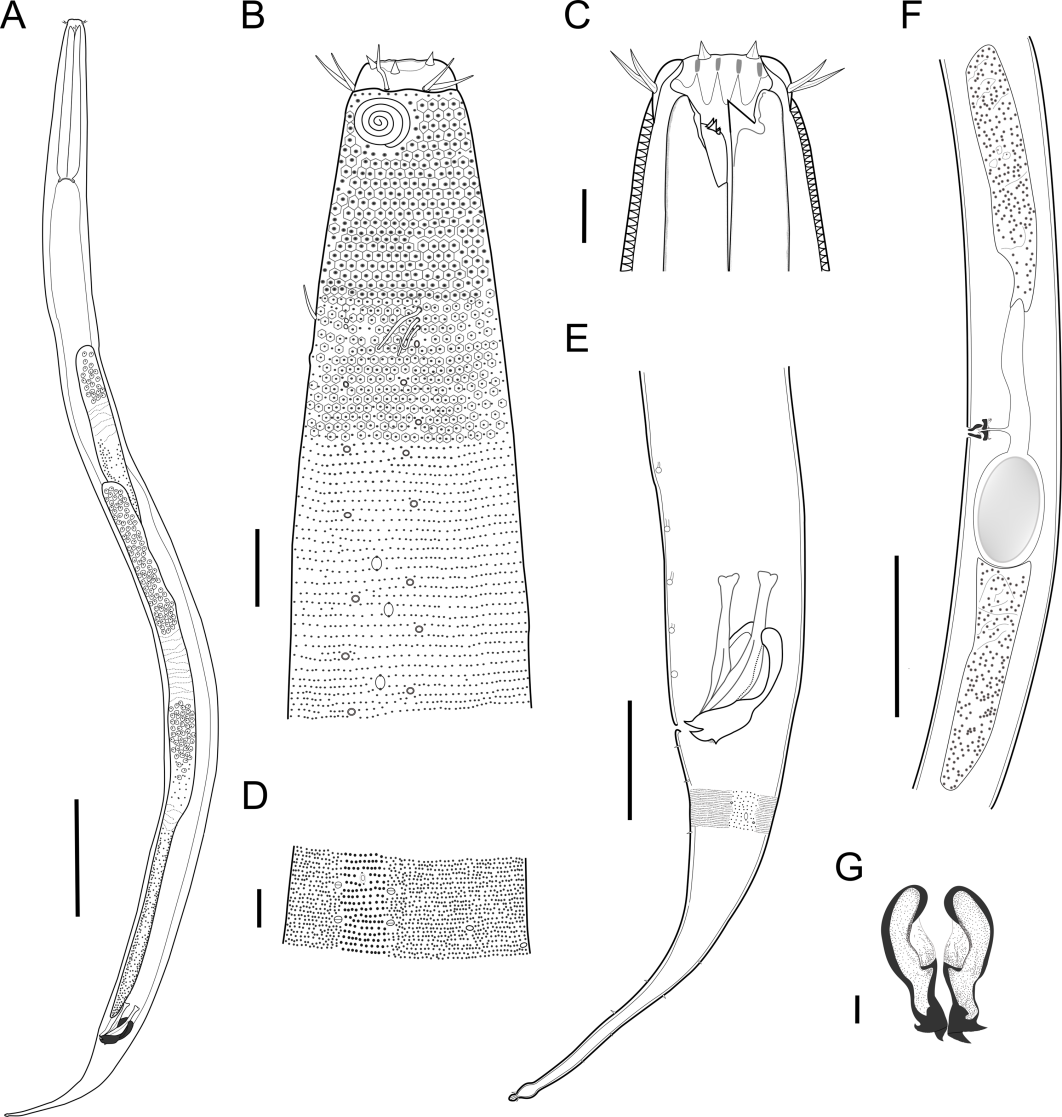
*Biarmifer nesiotes* sp. nov. (A) Entire body, male. (B) Anterior body region, male, superficial view. (C) Male head. (D) Cuticle in middle body region, male. (E) Posterior body region, male. (F) Middle body region of a female showing the reproductive system. (G) Gubernaculum, ventral view. Scale bars: A = 200 µm; B = 20 µm; C, D, G = 10 µm; E = 50 µm; F = 100 µm.

**Figure 3:**
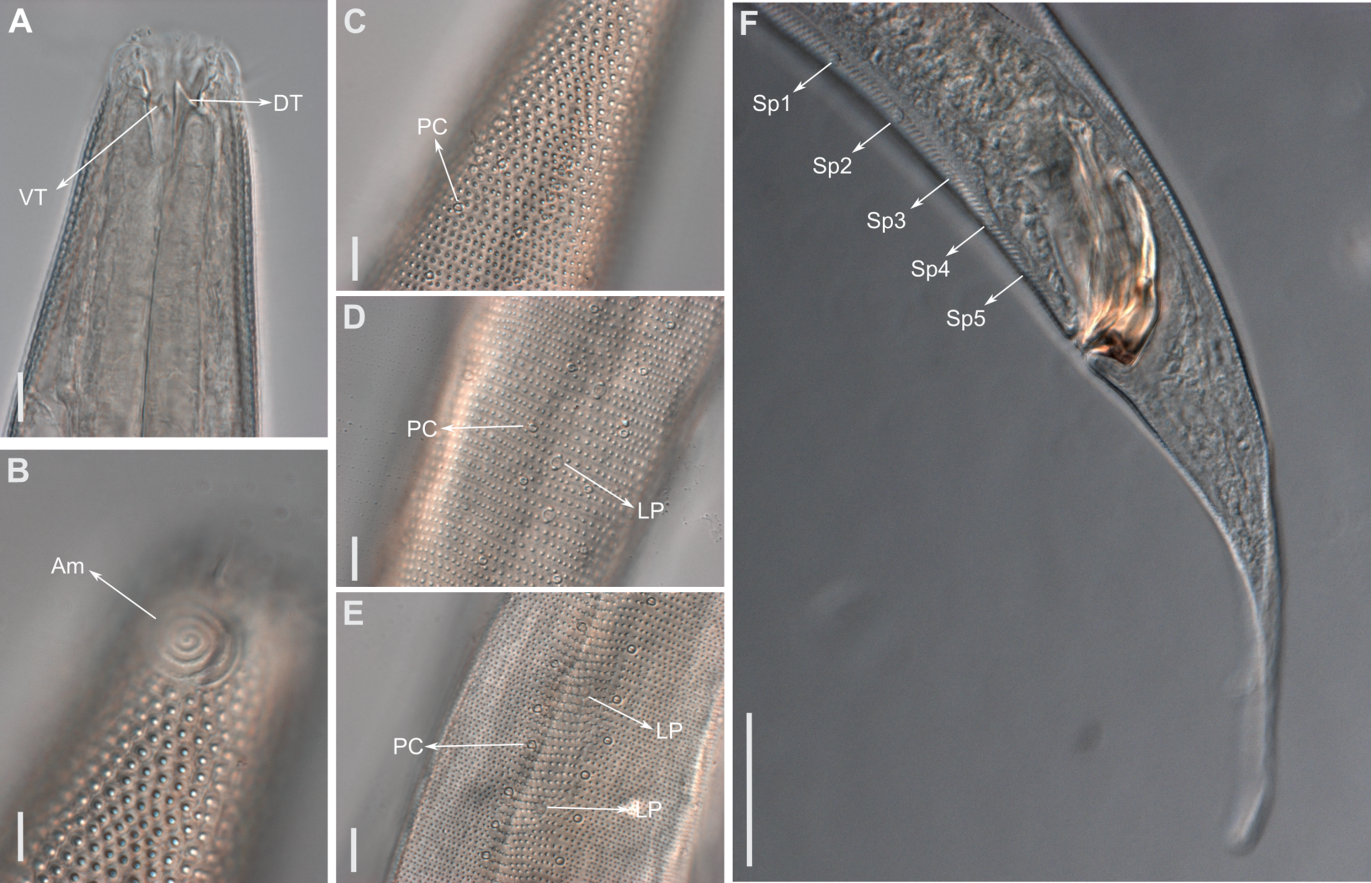
*Biarmifer nesiotes* sp. nov., light micrographs, male. (A) Anterior body region. (B) Detail of the multispiral amphideal fovea. (C) Cuticle from the anterior region to nerve ring. (D) Cuticle from the nerve ring to end of pharynx. (E) Cuticle from the middle body region. (F) Posterior region and copulatory apparatus. Am, amphideal fovea; DT, dorsal tooth; LP, like-pore structure; PC, pore complex; Sp, precloacal supplements; VT, ventrosublateral teeth. Scale bars A–E = 10 µm; F = 50 µm.

**Figure 4:**
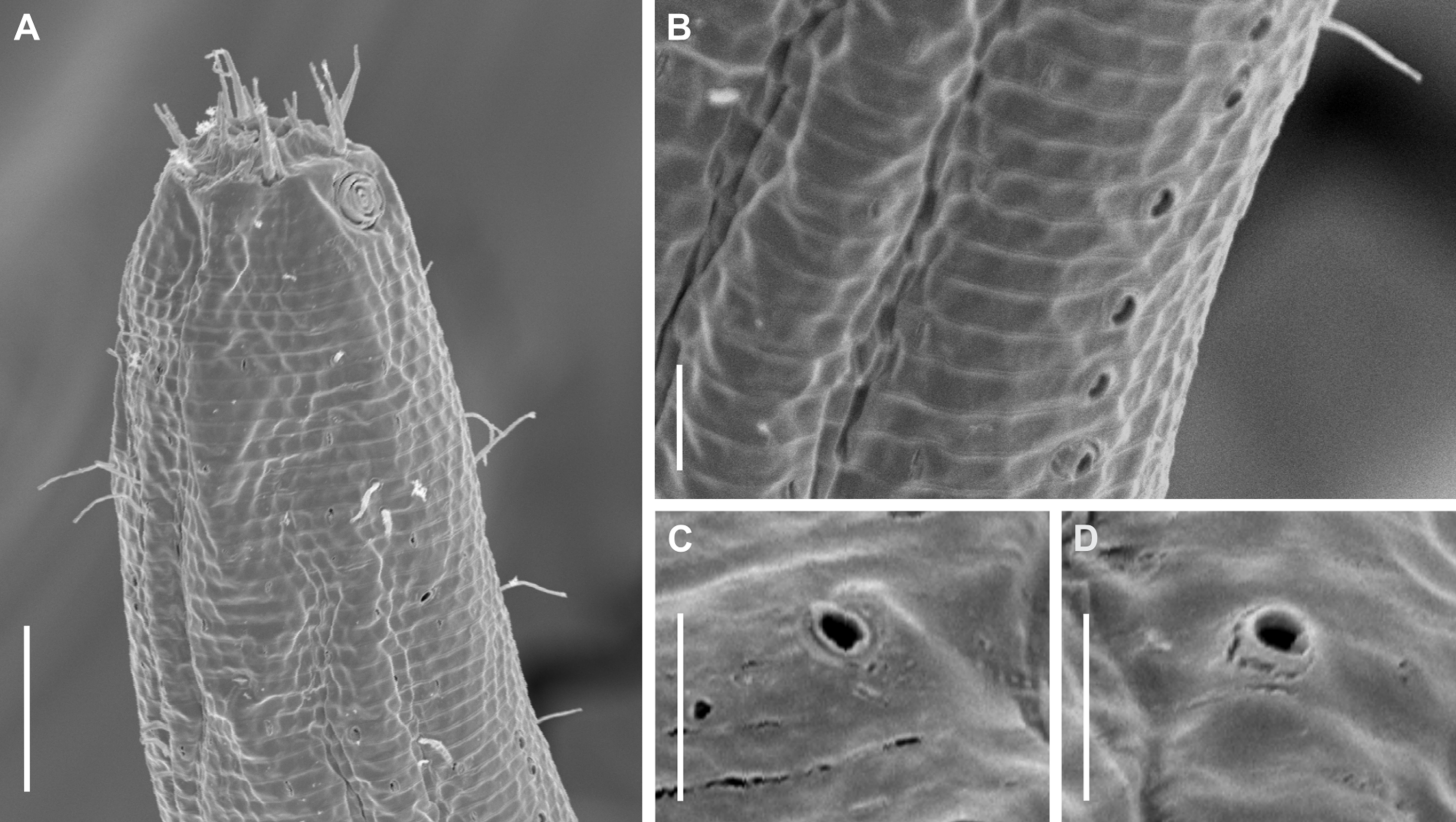
*Biarmifer nesiotes* sp. nov., scanning electron micrographs, male. (A) Anterior body region. (B) Anterior most lateral pore-like structures. (C) Detail of a pore complex in the precloacal region. (D) Detail of a lateral pore-like structure in the precloacal region. Scale bars: A = 20 µm; B–D = 5 µm.

**Figure 5:**
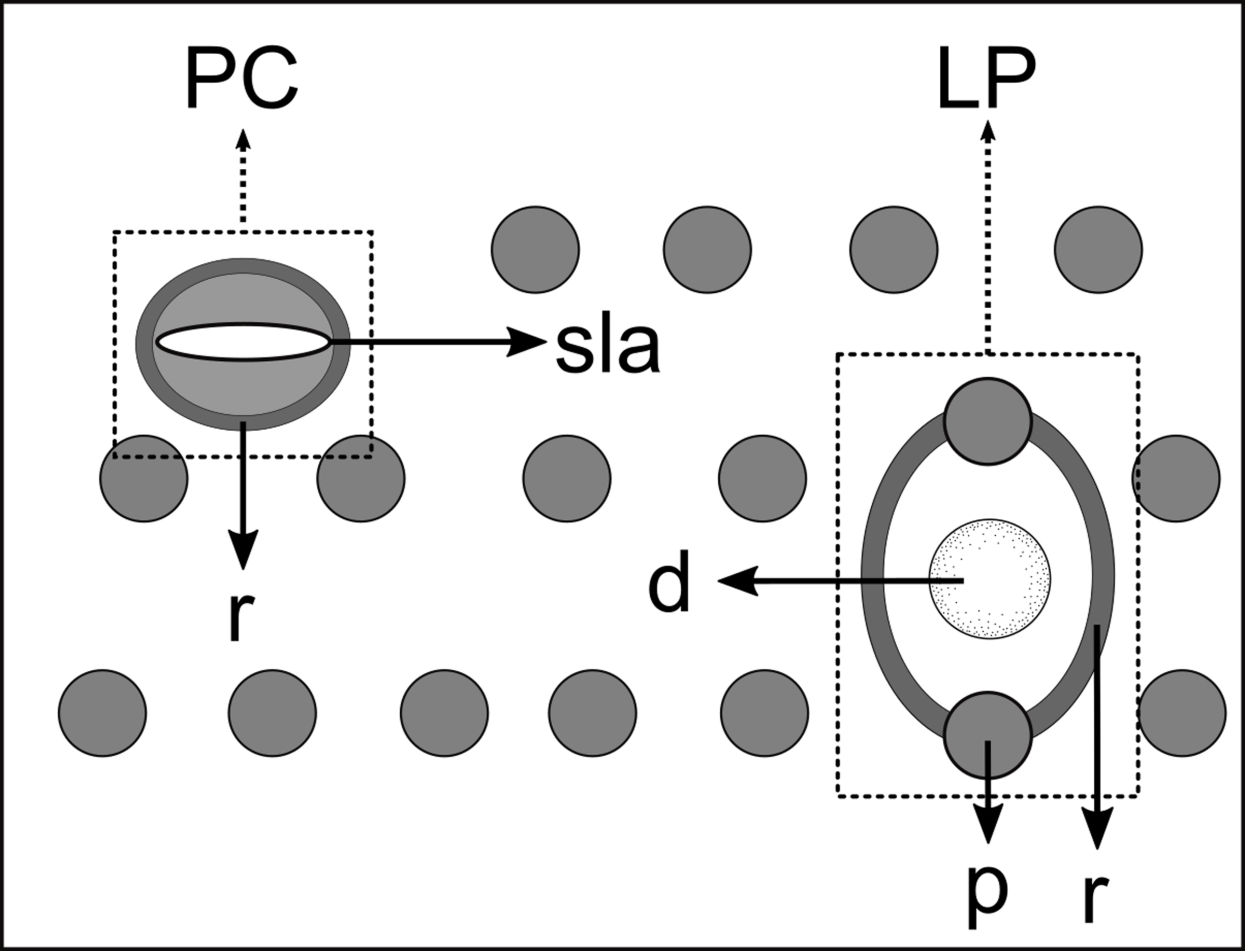
Schematic drawing of pore complex (PC) and lateral pore-like structures (LP) from the pharyngeal region of *Biarmifer nesiotes* sp. nov. d, dome; p, punctation; sla, slit-like aperture; r, cuticularized ring.

**Figure 6:**
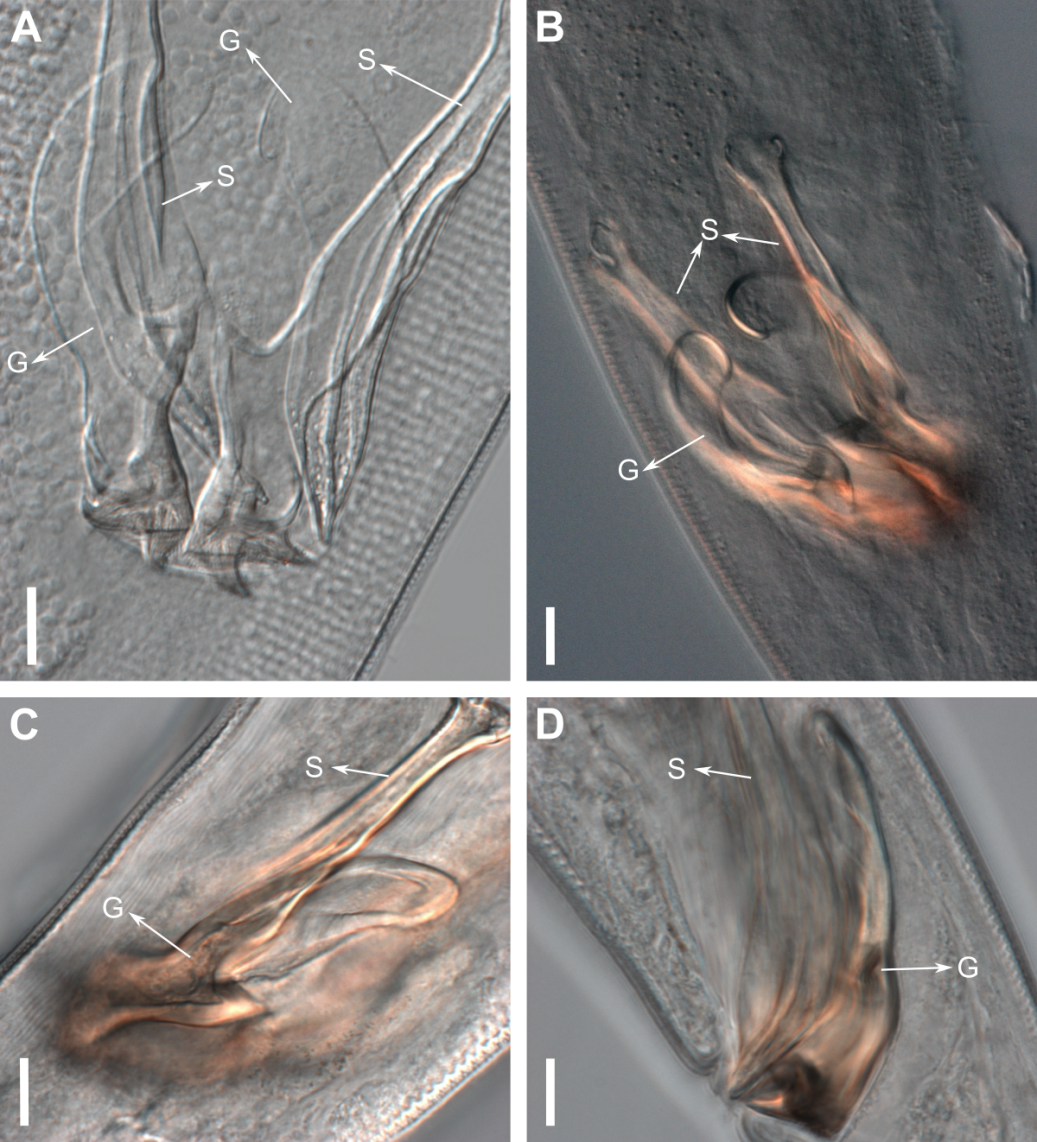
Gubernaculum in different positions, from different specimens of *Biarmifer nesiotes* sp. nov., lightning micrographs. (A) Ventral view. (B) Dorsal view. (C) Dorsolateral view. (D) Lateral view. G: gubernaculum; S: spicule. Scale bars: 10 µm.

**Figure 7:**
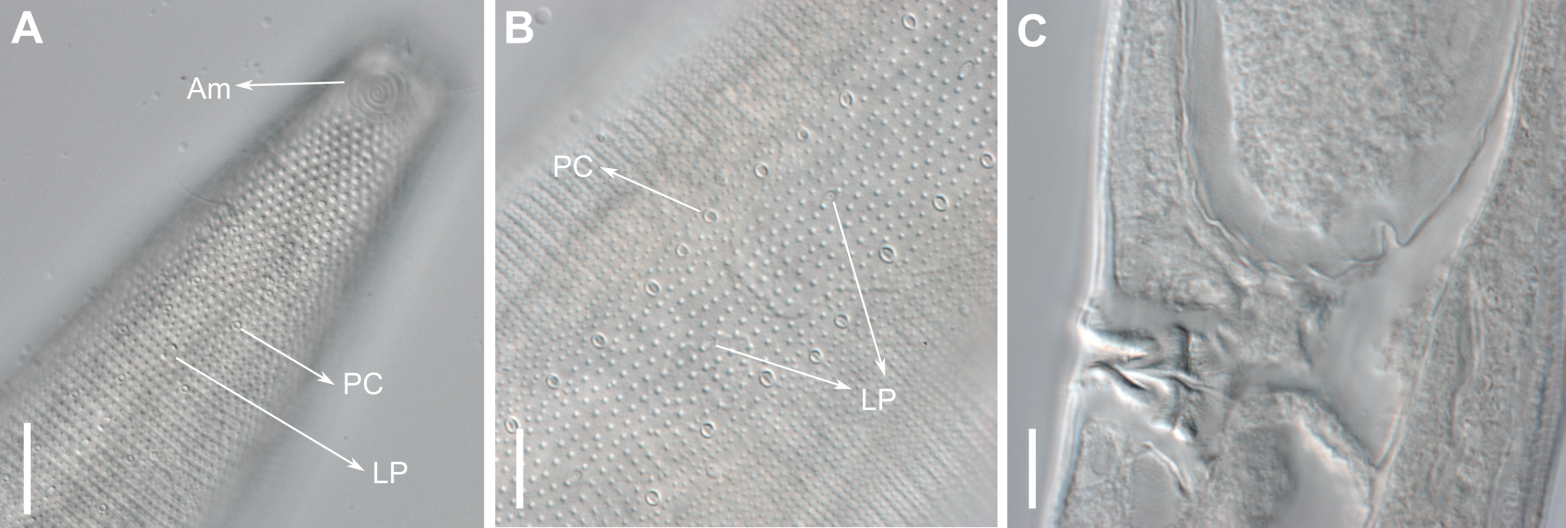
*Biarmifer nesiotes* sp. nov, lightning micrographs, female. (A) Anterior body region. (B) Cuticle from the middle body region. (C) Detail of the vulva and vagina. Scale bars: A = 20 µm; B–C = 10 µm.

***Biarmifernesiotes*sp. nov.** (Figs. 2–7, Table 1)
urn:lsid:zoobank.org:act:F5253EA9-84EA-4409-B033-34F53DDDCEE4

**Type locality:** Brazil, São Paulo State, São Sebastião Island, Pedras Miúdas beach (23°49′49″S, 45°23′27″W), subtidal zone, from sediment with gravel predominance

**Type specimens:** Holotype male (ZUEC-NMA 35, slide) and five males and seven females paratypes (ZUEC-NMA 36–43, slide), all from the type locality.

**Etymology:** The species name is derived from the Greek term *nesiotes* (=inhabitant of the island), as a reference to the type locality, São Sebastião Island.

**Description:** Holotype and paratype males. Body cylindrical, slightly narrower on the anterior extremity and tapering abruptly on the posterior end. Cuticle heterogeneous with bigger punctations surrounded by a hexagonal outline (Figs. 2B, 3B–3C) from the anterior sensilla to the nerve ring, and transverse rows of smaller, simpler punctations on the rest of the body (Figs. 2D, 3D–3E). Lateral differentiation consisting of larger and more spaced punctations, clearly visible from the posterior end of the pharynx to the tail tip. Lateral region with half the number of punctations rows when compared to the ventral and dorsal regions. Longitudinal row of lateral pore-like (LP) structures along each mediolateral line starting immediately after the excretory pore to the conical part of the tail; LPs consisting of a circular or elliptical opening supported by an unmodified punctation at the anterior and posterior extremities, with a central, non-cuticularized dome (Figs. 4B, 4D and 5); underlying gland cells absent. LPs more conspicuous and with a more cuticularized opening in the pharyngeal and tail region (∼2.7 × 2.2 µm in size) (Fig. 3D) when compared to the ones from the middle body region (∼1.9 × 1.7 µm) (Fig. 3E). Eight longitudinal rows of circular pore complexes, situated subventrally (2 rows), subdorsally (2 rows), and sublaterally (4 rows), starting about three head diameters from anterior end to the conical part of the tail; the pores are 1.2–1.9 × 1.3–1.9 µm in size and ∼4–16 µm a part, the sublateral ones more conspicuous; no differentiation in the distribution along the body. Pore complexes consisting of a cuticularized ring with a slit-like transversal oriented aperture, a few diagonally oriented (Figs. 4C and 5). Three somatic setae located on each sublateral axis on the pharyngeal region, 5 to 14 µm in length, at about 2.5 head diameter from the anterior end. Two longitudinal rows of setae on the tail.

Anterior sensilla arranged in two crowns; first crown consisting of six inner labial setae, 3 µm long (2.3–3.4 µm); second crown consisting of six slightly longer outer labial setae with a thinner tip (9–11.8 µm long) and four cephalic setae (6.5–8.5 µm long). Multispiral amphidial fovea with 5 turns and circular outline, corresponding to 50% of the cbd (46–50%), situated slightly posterior to the second crown of the anterior sensilla (Fig. 3B). Buccal cavity consisting of cup-shaped cheilostoma with 12 rugae and a narrow funnel-shaped pharyngostoma with a large dorsal tooth and two pairs of ventrosublateral teeth (Figs. 2C and 3A). Pharynx cylindrical, muscular and glandular. Secretory excretory system present; excretory pore located about $ \frac{1}{4} $ of the pharynx length; renette cell observed in one male, 41 × 18 µm in size. Cardia not surrounded by intestinal tissue. Intestinal lumen with numerous small golden-brown granules.

Reproductive system diorchic, gonads opposed. Spicules paired 83 µm long (72–83 µm), with prominences on the proximal end; fusiform in the distal half, dilated in the middle region, with a central groove, and pointed distal end (Fig. 6A). Gubernaculum complex (Fig. 6), proximally and distally paired (not fused), slightly shorter than the spicules, 65 µm long (60–70 µm), heavily cuticularized; forceps-shaped like in ventral or dorsal view (Fig. 2G); proximal end swollen and curved behind the spicules, mid-region with a lateral wing, distal end expanded with denticles. Five weakly developed cup-shaped precloacal supplements present, 17–27 µm apart from each other, the anterior-most at about 120 µm (88.2–122 µm) from the cloacal opening. Tail conical-cylindrical with the conical portion representing approximately 45% (Fig. 2E).

Paratype females. Similar to males but with slightly smaller amphidial fovea, corresponding to 40–45% of cbd and with four to five turns (Fig. 7A). Renette cell observed in one female, at the level of the posterior end of the pharynx and anterior end of the intestine, with 40 × 20 µm in size. Reproductive system didelphic, amphidelphic. Vagina and vaginal glands heavily cuticularized (Figs. 2F and 7C). Vulva located slightly pre-median. Up to four eggs observed per individual, 54–79 × 36–59 µm in size.

**Diagnosis.** *Biarmifer nesiotes* sp. nov. is characterized by a heterogeneous cuticle with lateral differentiation. Longitudinal rows of LP along mediolateral lines and eight longitudinal rows of pore complexes situated subventrally, subdorsally, and sublaterally. Outer labial setae about 10 µm long, and cephalic setae about 7 µm. Amphids with five turns (50% cbd) in males and 4–5 turns (40–45%) in females. Buccal cavity with one dorsal tooth and two pairs of ventrosublateral teeth. Spicules 72–83 µm long; gubernaculum 60–70 µm long, proximally and distally paired (not fused), proximal end swollen and curved behind the spicules, mid-region with a lateral wing, distal end expanded with denticles. Five weakly developed cup-shaped precloacal supplements. Vagina and vaginal glands strongly cuticularized.

**Figure 8:**
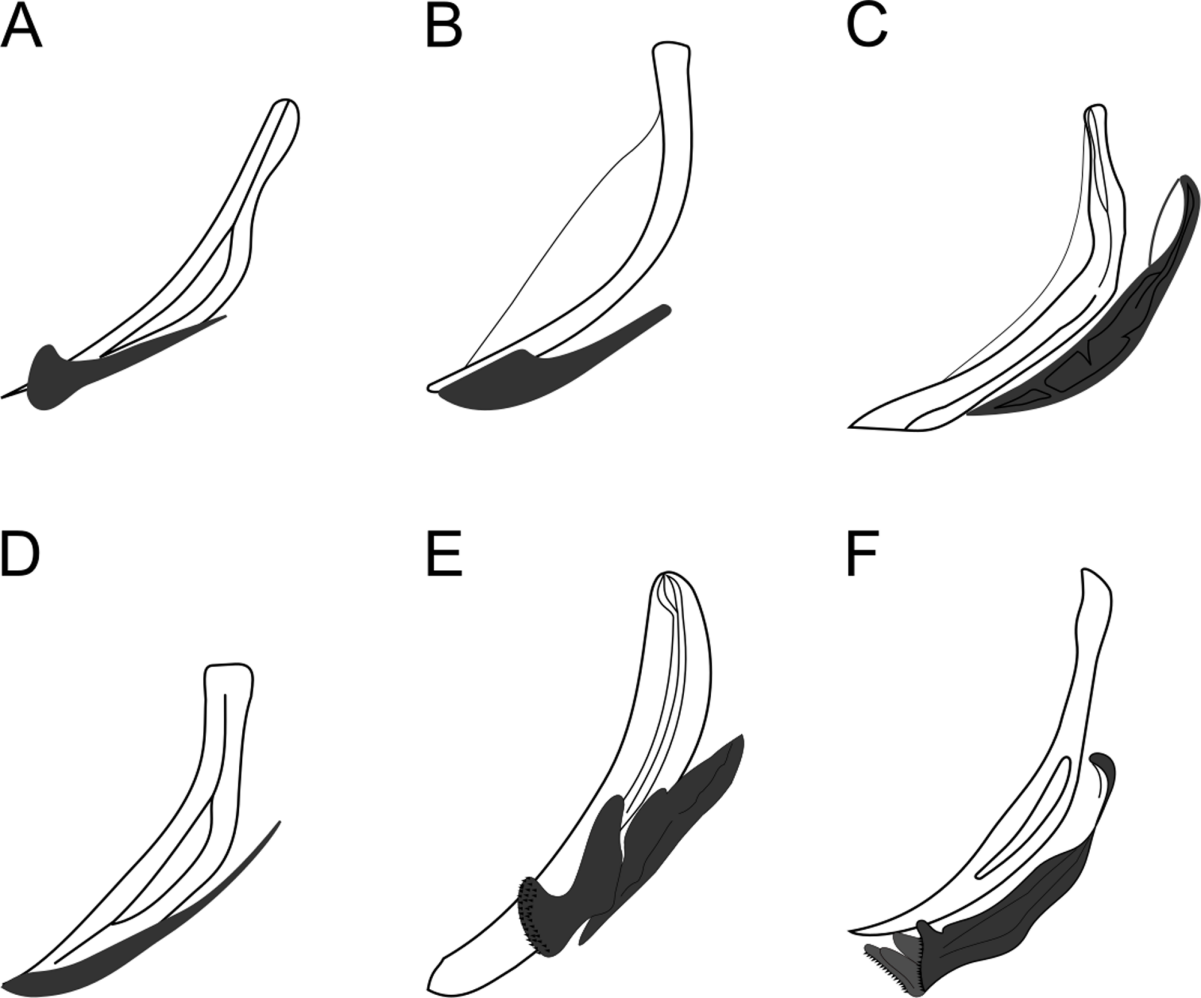
Copulatory apparatus of *Biarmifer* species. (A) *B. cochleatus*, adapted from Wieser (1954). (B) *B. dayi*, adapted from Inglis (1963). (C) *B. hopperi*, adapted from Sharma & Vincx (1982). (D) *B. laminatus*, adapted from Wieser (1954). (E) *B. madrynensis*, adapted from Pastor de Ward (2001). (F) *B. punctata*, adapted from Jensen (1985).

**Relationships:** *Biarmifer nesiotes* sp. nov. differs from all species of the genus by the shape of copulatory organs (Fig. 8). It also can be differentiated from the other six valid *Biarmifer* species by the combination of the following characters include in the tabular key of the genus (Table S1): lateral differentiation of cuticle; number of longitudinal rows of pore complex; amphidial turns; amphid width (as % of the corresponding body diameter); spicule length; number of precloacal supplements; and the De Man morphometric ratios (a, b, c, c′).

The new species is similar to *B. cochleatus* Wieser, 1954 in the number of supplements, conical-cylindrical tail and number of amphids turns, but differs by the smaller setae in the second crown of anterior sensilla (10 + 7 µm *vs*. 23–24 + 16 µm) and by the presence of lateral differentiation in the cuticle. The strong cuticularized vagina of females and the presence of eight longitudinal rows of pore complex in the cuticle (there are four rows in the other species) were observed only in the new species and in *B. hopperi* (Sharma & Vincx, 1982) Cunha, Fonseca & Amaral, 2022 among all species of the genus. It differs from the new species by the absence of lateral differentiation in the cuticle and by the shape of the copulatory apparatus. Also, *B. hopperi* has a cluster of 25–36 lateral pore-like structures in the mid-pharyngeal region, which are not observed in the new species (only 11–13 LP in the pharynx, located on the mediolateral line).

The aperture of pore complexes is transversally or diagonally oriented throughout the body of *Biarmifer nesiotes* sp. nov., different from *B. madrynensis* Pastor de Ward, 2001, where the aperture variated from diagonal (oriented 30° from the longitudinal axis) on pharynx and tail and longitudinal on the middle of the body (oriented 0° the from the longitudinal axis).

**Table:** 

Subfamily POMPONEMATINAE Gerlach & Riemann, 1973

**Diagnosis: (from Decraemer & Smol, 2006)** Body cuticle punctated, with lateral differentiation in ornamentation. Precloacal supplements knob-like or flattened, complex, consisting of several elements; gubernaculum paired proximally.

**Table:** 

Genus *Pomponema*Cobb, 1917

**Diagnosis:** **(modified from Cidreira et al., 2019)** Body cuticle heterogeneous with fine punctations, with or without lateral differentiation of longitudinal rows of enlarged punctations behind the cephalic region, and occasionally with slit-like markings. Cuticle in the cephalic region usually thick with punctations appearing in lateral view as elongated rods with Y-shaped ends. Inner labial sensilla setiform. Outer labial and cephalic sensilla setiform located in the same circle, usually jointed, with a tip section markedly narrower than the base, although this may be difficult to discern in some species. Outer labial setae larger than cephalic setae, but the four smaller setae are sometimes absent or at least so small and adherent to the outer labial setae that they cannot be detected. Amphidial fovea multispiral. Buccal cavity armed with a large pointed dorsal tooth, medium-sized ventrosublateral teeth, and with or without additional minute denticles. Pharyngeal bulb absent. Males with two opposed testes, rarely only one. Females didelphic-amphidelphic. Precloacal supplements complex, with several elements best seen in the ventral view. Gubernaculum with variously structured distal ends, sometimes with L-shaped lateral plates or lateral flanges bearing blunt teeth. Tail conical-cylindrical with a swollen tip.

**Remarks:** The species *Bolbolaimus punctata* Cobb, 1920 was transferred to the *Pomponema* genus by Luc & De Coninck (1959); however, it was re-established as *Bolbolaimus* species by Jensen (1978).

**Table:** 

**Figure 9:**
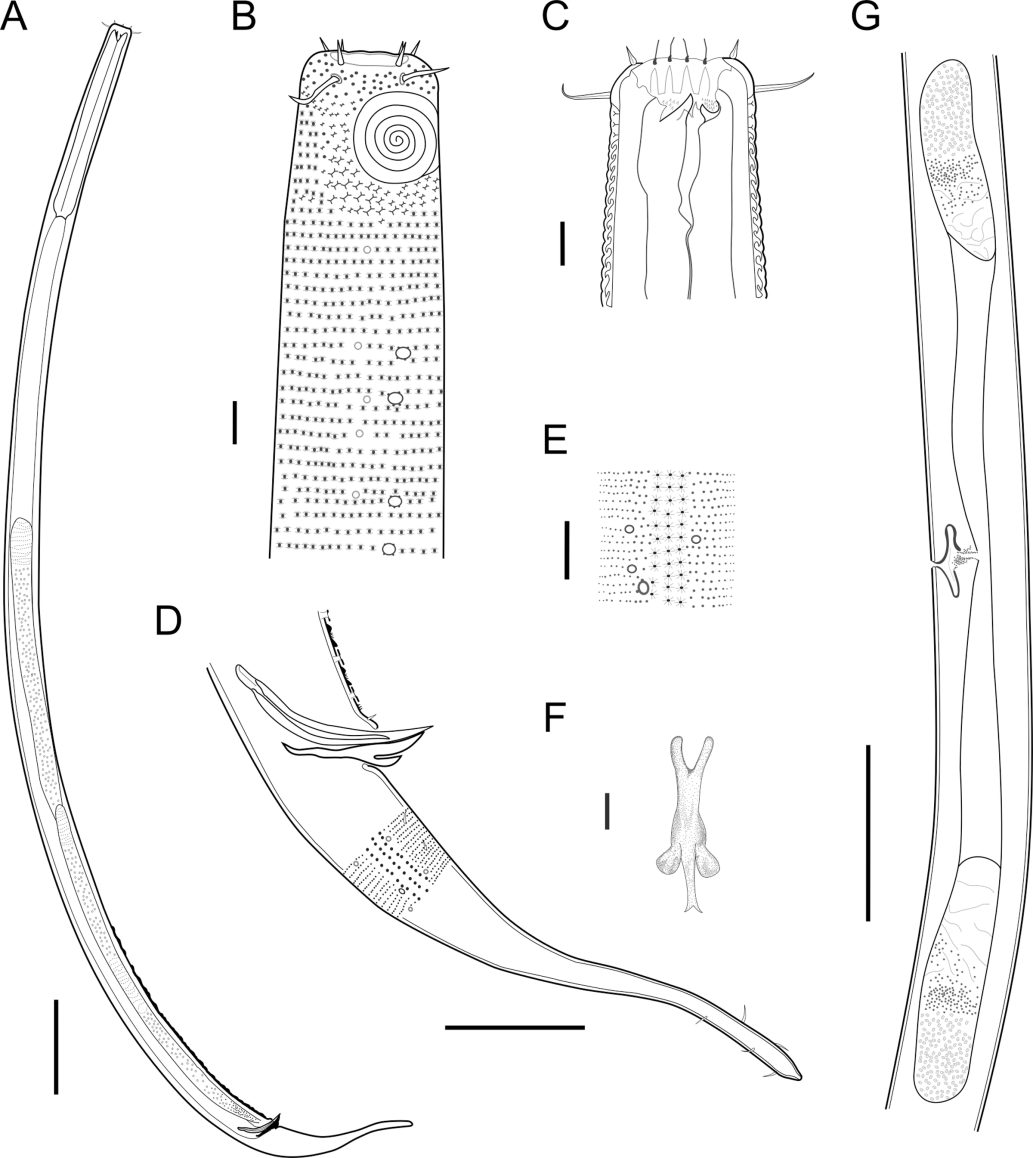
*Pomponema longispiculum* sp. nov. (A) Entire body, male. (B) Anterior body region, male, superficial view. (C) Cephalic region, male. (D) Posterior body region, male. (E) Cuticle in the mid-body region, male. (F) Gubernaculum, ventral view. (G) Middle body region of a female showing the reproductive system. Scale bars: A = 200 µm; B, C, E, F = 10 µm; D = 50 µm; G = 100 µm.

**Figure 10:**
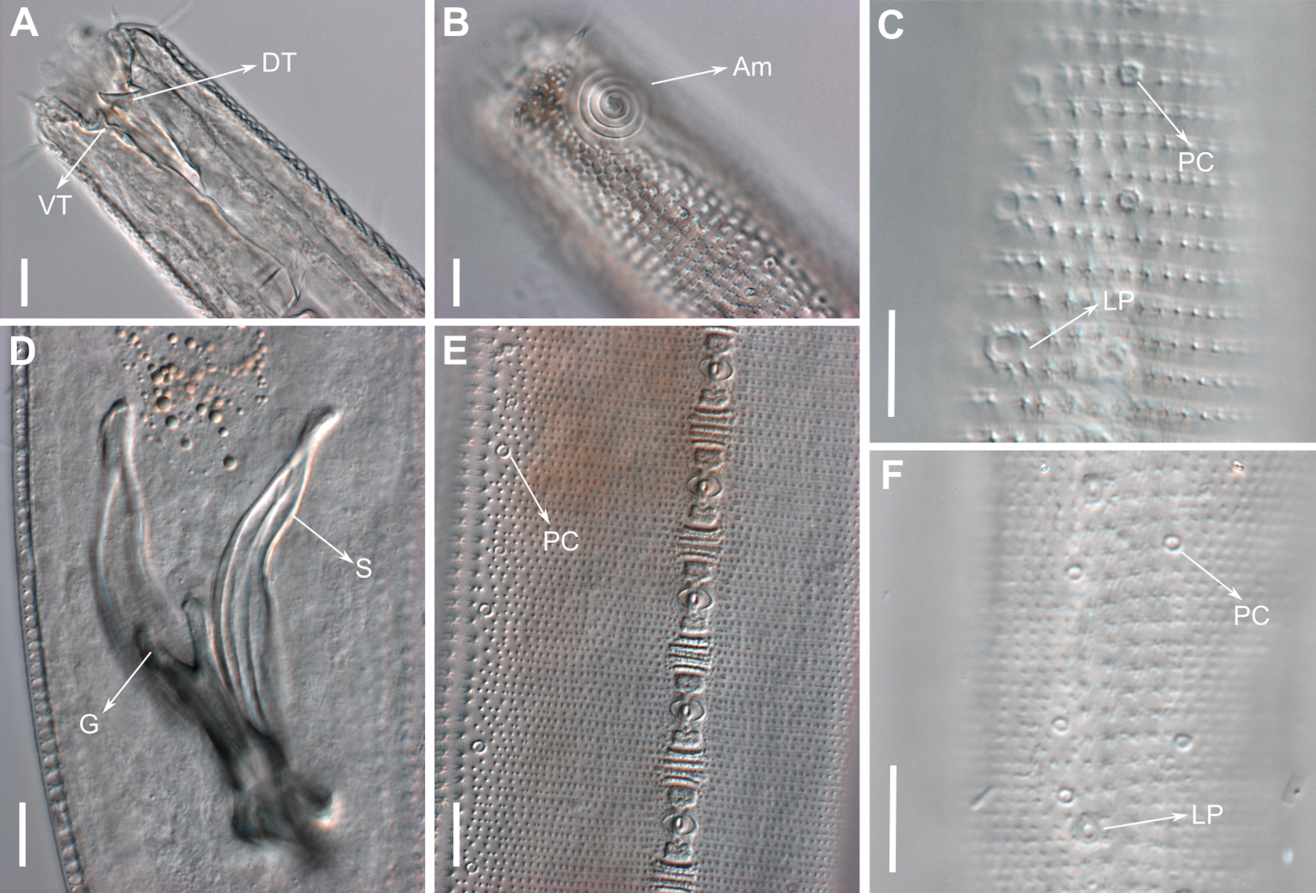
*Pomponema longispiculum* sp. nov., lightning micrographs, male. (A) Anterior body region. (B) Surface view of the anterior body region. (C) Cuticle in the pharyngeal region. (D) Copulatory apparatus, ventral view. (E) Detail of precloacal supplements in ventral view. (F) Cuticle in the middle body region. Am, amphideal fovea; DT, dorsal tooth; G, gubernaculum; LP, lateral pore-like; PC, pore complex; S, spicule; VT, ventral teeth. Scale bars = 10 µm.

**Figure 11:**
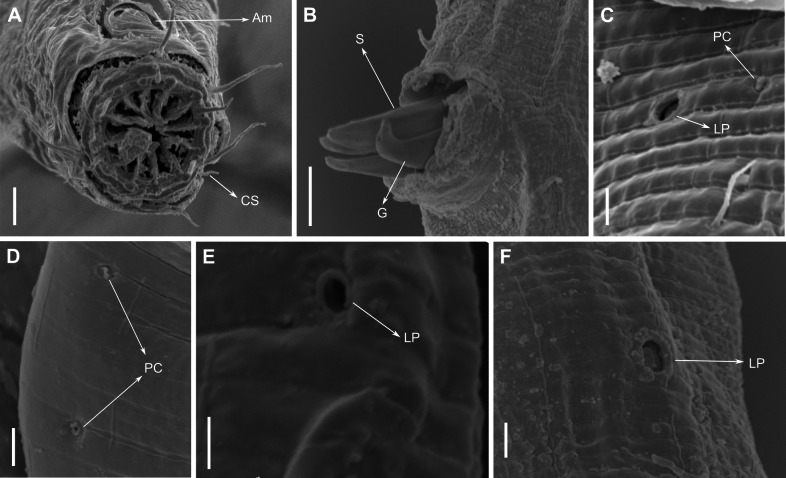
*Pomponema longispiculum* sp. nov., scanning electron micrographs, male. (A) Head in frontal view. (B) Cloacal region, showing the distal ends of the copulatory apparatus. (C) Like-pore structure and pore complex in the anterior region. (D) Pore complexes in the middle body region. (E) Like-pore structure in the middle body region. (F) Like-pore structure in the tail. Am, amphideal fovea; CS, cephalic seta; G, gubernaculum; LP, lateral pore-like; PC, pore complex; S, spicule. Scale bars: A, B = 5 µm; C, D, F = 2 µm; E = 1 µm.

**Figure 12:**
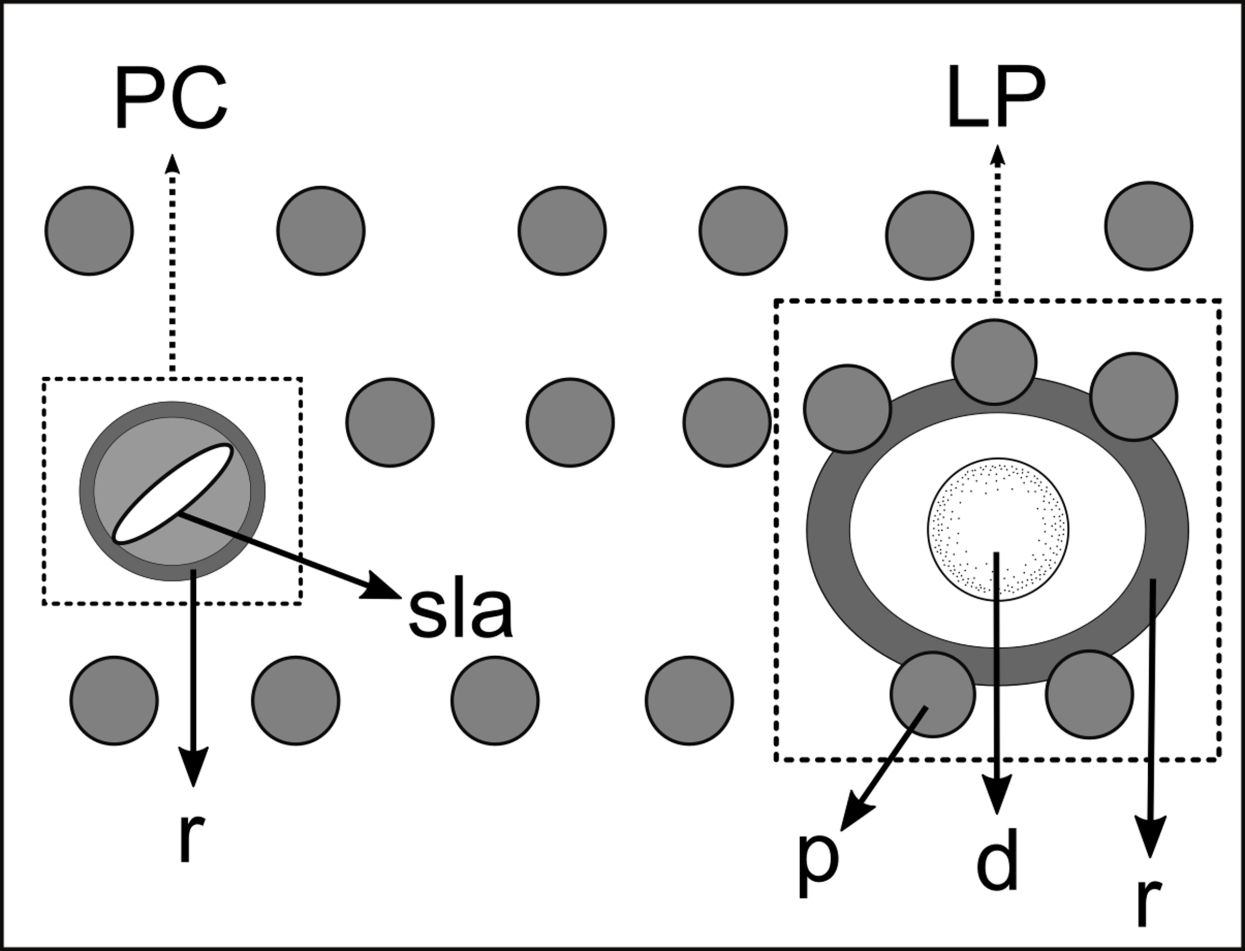
Schematic drawing of pore complex (PC) and lateral pore-like structures (LP) from the pharyngeal region of *Pomponema longispiculum* sp. nov. d, dome; p, punctation; sla, slit-like aperture; r, cuticularized ring.

***Pomponema longispiculum*sp. nov.** (Figs. 9–12, Table 1)
urn:lsid:zoobank.org:act:AE58C8EA-4EF9-4ED7-9265-AAA07EFEBCBA

**Type locality:** Brazil, São Paulo State, São Sebastião Island, Pedras Miúdas beach (23°49′49″S, 45°23′27″W), subtidal zone, from sediment with gravel predominance.

**Table 1:** Morphometric data of the two new species.

	*Biarmifer nesiotes* **sp. nov.**			*Pomponema longispiculum* **sp. nov.**		
	Male Holotype	Males Paratypes	Females Paratypes	Male Holotype	Males Paratypes	Females Paratypes
N	–	5	7	–	3	5
L	1871.5	1,252–1,744	1,488–1,747	1854.4	1,352–1,684.8	1,396–1855.5
a	23.3	17.4–26.6	17.3–25.7	39	30.4–39.7	28–36.5
b	6.8	5.8–6.8	5.8–6.8	7	6.6–7	5.7–6.6
c	10.5	7.3–8.1	7–8.9	9.6	7.4–8	6–8.4
c′	3.5	3.6–4.2	4.6–5.6	4.7	5.5–5.8	6.1–7.5
Pharynx length	276.6	215.3–285.7	235.5–283.5	265.6	206–242	244–285.2
Pharyngeal diam. at base	32	24.4–28.3	22.3–32.3	25	17–20	18.3–26.7
Pharynx cbd at base	74	52–81.6	49.3–80.7	43.3	41–44	39–47.3
Max. body diam.	80.4	60–100	59–101	47.4	34–51	45–58
Tail length	177.7	171.8–220.3	167–242	193.5	176–211.5	199.3–233.2
Length of inner labial setae	3	2–3.4	2.4–3.5	5.4	4–6.4	4.6–6.5
Length of outer labial setae	10.5	9.1–11.8	9.3–12	14	12–15	11.5–14.6
Length of cephalic setae	7.7	6.5–8.5	7–9.7	4.8	2.8–4.2	3.3–3.6
Head diam. at cephalic setae	24.8	24–25	24.3–25.7	33	29–34	32.5–35
Head diam. at amphids	30	27–30.5	27–30.8	33.6	30–32.7	34.3–37
Amphid turns	5	5	4–5	5.5	6	4–5
Amphid height	13	12.3–14	8.8–12.7	21,76	15.4–19	10.5–12
Amphid width	15	12.8–14.7	11–13.5	19.2	17–19.5	10.8–11.3
Amphid width/cbd (%)	50	47–48.2	40–46	57	54.8–59.6	29.2–32.9
Amphid from anterior end	9.7	7.5–10.8	6–9.2	11.2	8.1–10.7	6.6–10
Excretory pore from anterior end	75.4	61–70	68.7–77.6	–	127^a^	–
Nerve ring from anterior end	119.7	114–118	116–123.2	120.6	110.8–113	116–130.4
Nerve ring cbd	60	54–57	54–57	40.3	35–41	–
Spicule length	82,6	72–83.7	–	75.7	72.5–78	–
Gubernaculum length	65	60–70	–	49.6	44–47.8	–
V	–	–	731–847	–	–	780–996.8
% V	–	–	47–49	–	–	52.1–55.9
Vulval cbd	–	–	68.5–99.2	–	–	43.3–54
Abd	51	47–59	36–48.4	41.4	32–38.4	30–37.3
Laterodorsal PC (pharynx)	23	18–22	15–21	13	9	13–17
Laterodorsal PC (central body)	145	114–129	108–155	77	75	60–61
Laterodorsal PC (tail)	5	6–8	5–9	7	6	5–7
Lateroventral PC (pharynx)	25	15–27	13–20	18	8–15	10–17
Lateroventral PC (central body)	138	118–141	116–139	71	60–61	55–69
Lateroventral PC (tail)	7	6–8	6–8	7	4	5–7
LP (pharynx)	11	11–13	11–16	5	5–6	5–7
LP (central body)	45	38–52	30–40	29	29–33	26–41
LP (tail)	3	3–5	5–8	2	2–3	2

**Notes.**

a Observed in only one specimen.

Abbreviations a body length/maximum body diameter abd anal body diameter b body length/pharynx length c body length/tail length c′ tail length/anal or cloacal body diameter cbd corresponding body diameter L total body length LP lateral pore-like structures N number of specimens PC pore-complexes V vulva distance from anterior end of body %V position of vulva as a percentage of body length from anterior end

**Type specimens:** Holotype male (ZUEC-NMA 50, slide) and three male and five female paratypes (ZUEC-NMA 51–58, slide), all from type locality.

**Etymology:** The species name is derived from the Latin words *longis* (= big, long) and *spiculum* (=spicule), and refers to the long spicules of this species in comparison to the other species of the genus.

**Description:** Holotype and paratype males. Body cylindrical, slender (Fig. 9A). Cuticle heterogeneous; thicker in the cephalic region, with punctations appearing in the lateral view as Y-shaped. Transverse rows of larger punctations on the anterior region of the body, gradually decreasing posteriorly. Lateral differentiation consisting of two to four longitudinal rows of punctations, beginning approximately at $ \frac{3}{4} $ of the pharynx length from the anterior end. Lateral pore-like structures (LP) consisting of a circular or elliptical strongly cuticularized and thick opening supported by unmodified punctations and with a central, non-cuticularized dome (Figs. 10C, 10F; 11C, 11E, 11F and 12); underlying gland cells absent. Four to five conspicuous lateral pore-like structures (LP), 2–3 × 2.7–3.5 µm in size, located at mediolateral lines between amphidial fovea and the nerve ring, beginning approximately at two head diameters from the anterior end; opening supported by four to six punctations (Figs. 10C and 12). From the beginning of the lateral differentiation to the conical part of the tail, the LPs are slightly smaller, 1.5–2.8 × 1.5–2.6 µm in size, and located between the mediolateral and laterodorsal lines (Figs. 9E and 10F), their opening is supported by two to four unmodified punctations. Four sublateral longitudinal rows of circular pore complexes, ∼1–1.8 × 1.2–1.9 µm in size and ∼6–17 µm apart from each other, consisting of a cuticularized ring with a slit-like aperture, arranged 30° of the longitudinal axis (Fig. 12). Few somatic setae located sublaterally on the pharyngeal region (7–15 µm long) and four sublateral longitudinal rows of small setae along the body, sometimes difficult to differentiate from the pore complexes.

Anterior sensilla arranged in two crowns; first crown consisting of six inner labial setae, 5.4 µm long (4–6.4 µm); second crown consisting of six outer labial setae with a thinner tip, 14 µm long (12–15 µm), and four inconspicuous cephalic setae adherent to the outer labial setae (Fig. 11A), 4.8 µm long (2.8–4.2 µm). Multispiral amphidial fovea with 5.5–6 turns and circular in outline, representing 57% of the corresponding body diameter (54.8–59.6%), situated slightly posterior to the second crown of the anterior sensilla (Figs. 9B and 10B). Buccal cavity cup-shaped, cheilostoma with 12 rugae and a narrow funnel-shaped; pharyngostoma with a large dorsal tooth, two pairs of ventrosublateral teeth and three to four rows of denticles (Fig. 9C). Cylindrical pharynx, slightly enlarged anteriorly. Secretory excretory system observed in only one male; excretory pore located approximately at the middle pharynx, renette cell 69 × 20.4 µm long located after the end of the pharynx. Cardia not observed. Intestinal lumen with numerous small golden-brown granules.

Reproductive system diorchic, gonads opposed. Spicules arched with a central lamella (Fig. 10D), 75.7 µm long (72.5–78 µm), corresponding to 1.8 times (1.9–2.4) the cloacal body diameter. Gubernaculum fused, with lateral wings and distal end forked, bearing a pore in each tip (Figs. 9F, 10D and 11B). There is a short precloacal seta, ∼3 µm long. There are 22 (19–21) precloacal supplements extending 365 µm (256–307 µm) anteriorly from the cloaca. Supplements are composed of an outer plate, H-shaped in ventral view, with a pore running through the center (Fig. 10E). Above this plate, there is an elliptical cuticularized ring. Anteriorly and posteriorly of each supplement there is a cuticularized element of irregular shape. There are 4 to 7 bars (lamellated cuticle) between each supplement. Tail conical-cylindrical (Fig. 9D), corresponding to 4.7 times the anal body diameters.

Paratype Females. Similar to males but with a smaller amphidial fovea (5.5–6 turns and 54.8–59.6% cbd in males *versus* 4–5 turns and 29.2–32.9% in females). Reproductive system didelphic, amphidelphic with the anterior reflexed ovary lying on the left of the intestine and the posterior reflexed ovary lying on the right of the intestine (Fig. 9G). No mature eggs observed. Vulva located slightly post-median body region.

**Diagnosis**: *Pomponema longispiculum* sp. nov. is characterized by a heterogeneous cuticle with lateral differentiation consisting of two to four longitudinal rows of punctations. Four to five conspicuous lateral pore-like structures between the amphidial fovea and nerve ring and a longitudinal row of smaller LPs between the mediolateral and laterodorsal lines, from the beginning of lateral differentiation to the tail. Four longitudinal rows of pore complexes situated sublaterally. Six outer labial setae 12–15 µm long, and four inconspicuous cephalic setae, 2.8–4.8 µm long. Amphids with 5.5–6 turns (55–60% cbd) in males and 4–5 turns (29–33% cbd) in females. Buccal cavity with one dorsal tooth, two pairs of ventrosublateral teeth, and three to four rows of denticles. Spicules with lamella, with length corresponding to 1.8–2.4 times the anal cbd; 19–23 typical precloacal supplements; and gubernaculum 44–50 µm long, fused.

**Relationships:** *Pomponema longispiculum* sp. nov. can be differentiated from the other 31 valid *Pomponema* species (Cunha, Fonseca & Amaral, 2022) by the following characters included in the tabular key (Table S2): lateral differentiation of cuticle; presence or absence of ventral teeth; presence or absence of rows of denticles in the buccal cavity; amphid width (as % of the corresponding body diameter); amphidial turns; length of outer labial setae; spicules length; number of precloacal supplements; and De Man morphometric parameters (a, b, c, c′).

*Pomponema longispiculum* sp. nov. is similar to *P. stomachor* Wieser, 1954, which was described from the Chilean coast. The new species differs by the relative size of amphidial fovea (55–60% cbd in males and 30% in females *vs.* 50% in both sexes in *P. stomachor*), the shorter tail in males (4.7–5.8 *vs*. 7 cloacal diameters in length), the shorter labial sensilla in males (inner labial setae 4–6 µm long *vs*. 9 µm; and outer labial setae 12–15 µm long *vs*. 18 µm), the presence of two pairs of subventral teeth *vs*. one pair in *P. stomachor*, and the beginning of the cuticle lateral differentiation ($ \frac{3}{4} $ *vs.* end of the pharynx). Both species have spicules of similar length, however, it represents 1.8–2 anal diameters in *P. longispiculum* sp. nov. and 1.7 in the Chilean one. The shape of the gubernaculum is not described in detail and is not clearly visible on drawings of *P. stomachor*. The new species is also similar to *P. reductum* Warwick, 1970 regarding the gubernaculum shape, but they differ by the length of outer labial setae and the spicules (see Table S2), and by the cuticle ornamentation. In *P. reductum* the cuticle has smaller irregularly arranged punctations in the anterior region which gradually increase in size towards the lateral fields. *Pomponema longispiculum* sp. nov. has bigger punctations in the anterior region and punctations in the lateral field of the same size throughout the body.

**Figure 13:**
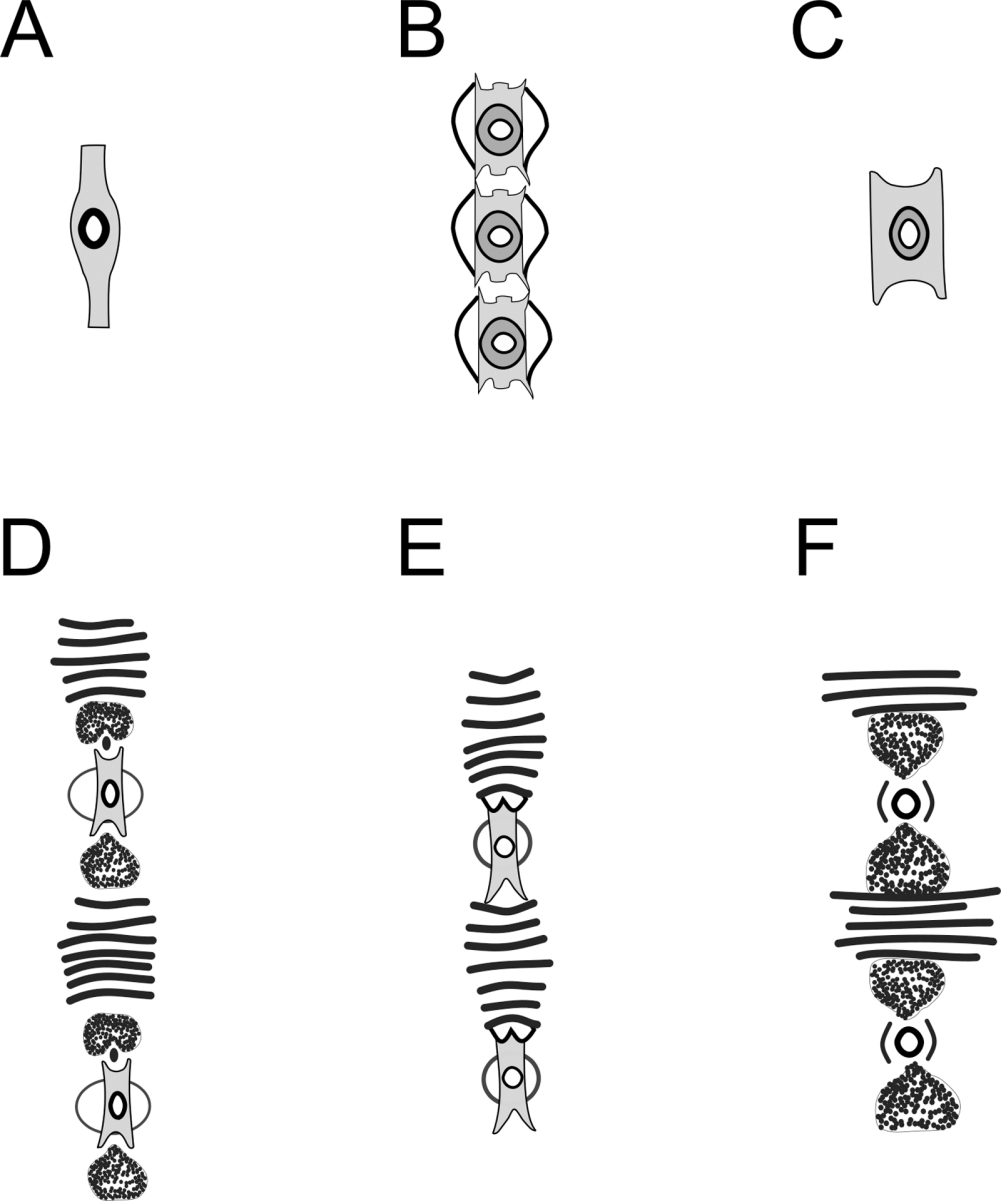
Schematic drawing of precloacal supplements from *Pomponema* species, in the ventral view. (A) *P. concinnum*, adapted from Wieser (1954). (B) *P. corniculata*, adapted from Gourbault (1980). (C) *P. hastatum*, adapted from Ott (1972). (D) *P. longispiculum* sp. nov. (E) *P. sedecima*, adapted from Platt (1973). (F) *P. tesselatum*, adapted from Platt & Warwick (1988). Note: The bars of lamellated cuticle are not represented by Ott in ventral view, but they are present in *P. hastatum*. However, the bars seem to be absent in *P. concinnum* and *P. corniculata*.

The general aspect of precloacal supplements from *P. longispiculum* sp. nov., *P. sedecima* and *P. tesselatum* Wieser & Hopper, 1967 are similar (Fig. 13). These structures differ among those species in the number of bars between each supplement, the absence of cuticularized elements of irregular shape in *P. sedecima*, and the absence of an outer plate, H-shaped in ventral view, in *P. tesselatum*.

*Pomponema tesselatum* is the only species of the genus that presents detailed descriptions of the pore complexes and LPs structures. According to Hopper (1972), this species has the aperture of pore complexes longitudinally oriented and only 7–18 LPs throughout the body. In *P. longispiculum* sp. nov. the slit-like aperture is diagonally oriented and there are more LPs throughout the body (34–49).

Based on the SSU molecular sequence deposited in the Genbank, *Pomponema longispiculum* sp. nov. is closest to *Pomponema* sp. (MN250093) with 8.73% of difference (64 in 733 bp). The next two most similar sequences recovered are from *Praeacanthonchus punctatus* (MG669976 and MG669981), with 67 and 68 bases pairs different, respectively.

## Discussion

Prior to the present study, seven cyatholaimid species were described from Brazil, most of them in the 1950s (Gerlach, 1957a; Gerlach, 1957b; Oliveira et al., 2017; Cidreira et al., 2019). *Biarmifer nesiotes* sp. nov. is the first species of the genus recorded from the country. Five species of *Pomponema* were recorded in Brazilian coastal habitats so far (Venekey, Fonseca-Genevois & Santos, 2010; Venekey, 2017; Cidreira et al., 2019): *P. corniculata* Gourbault, 1980; *P. cotylophorum* (Steiner, 1916) Lorenzen, 1972; *P. sedecima* Platt, 1973; *P. tautraense* (Allgén, 1933) Lorenzen, 1972 and *P. veronicae* Cidreira et al., 2019, this last one described for Itapuã beach located on the Northeastern Brazilian coast. The records of *P. cotylophorum* and *P. sedecima* from Brazil (Silva, 2012) must be taken with caution since the specimens found have morphological differences from the original descriptions that could be considered interspecific variations. With the present work, the number of cyatholaimids registered in Brazil raised to 14 genera and 22 species.

### Diagnostics characters within Cyatholaimidae

The principal issues in the Cyatholaimidae taxonomy are the relative aspect of the weight given to each character and the lack of detailed descriptions of morphological structures that may be taxonomically relevant (Cunha, Fonseca & Amaral, 2022). The pattern of ornamentation of the cuticle is very important for the delimitation of the *Biarmifer* genus, for example. However, for *Pomponema* identification, the buccal cavity and the precloacal supplements are the principal diagnostic characters, whereas the ornamentation of the cuticle is very variable between the species (see Table S2).

Wieser (1954) first erected the *Biarmifer* genus based on the presence of double spicules, with a median lacuna. Pastor de Ward (2001) described the spicules of *B. madrynensis* as having inner septa and updated the diagnosis of the genus as “spicules with inner processes”. The researcher also noted the typical configuration of the cuticle in all species of the genus and emended the diagnosis. Here we considered the cuticle ornamentation as the main diagnostic character, since this is unique to the genus, and due to the lack of an accurate description of the spicules. In fact, all *Biarmifer* species described so far seem to have some kind of inner processes in the spicules, but they are variable. In *B. cochleatus* and *B. laminatus* Wieser, 1954 the spicules present a median lacuna, whereas the spicules of *B. dayi* (Inglis, 1963) Cunha, Fonseca & Amaral, 2022 and *B. punctata* (Jensen, 1985) Cunha, Fonseca & Amaral, 2022 have alae. The drawings of *B. hopperi* suggest that its spicules also have inner processes (Sharma & Vincx, 1982). Accordingly, to the micrographs of *B. madrynensis* provided by Pastor de Ward (2001), instead of the inner septa described, the spicules seem to be similar to that from *Biarmifer nesiotes* sp. nov. with a central groove.

The supplements of *Pomponema*, as well as of *Craspodema*, are large and complex, formed by several elements and with the cuticle lamellated between them (type A *sensu* Wieser & Hopper, 1967). These complex structures may vary among species and ventral view observations reveal details that are not visible otherwise. However, until now, only the descriptions of five valid *Pomponema* species included illustrations of supplements ventral view: *P. concinnum* (Wieser, 1954) Lorenzen, 1972, *P. corniculata* Gourbault, 1980, *P. hastatum* (Ott, 1972) Cidreira et al., 2019, *P. sedecima* Platt, 1973, *P. tesselatum* Wieser & Hopper, 1967 and *Pomponema longispiculum* sp. nov (see Fig. 13). Besides the variation in the number of bars between the supplements, the shape of the outer plate is very diverse. An elliptical cuticularized ring above this plate is present in *P. corniculata*, *P. sedecima*, and *P. longispiculum* sp. nov., absent in *P. concinnum* and *P. hastatum*, and incomplete in *P. tesselatum*. Other elements, such as irregular-shaped structures, are present between the supplement and the cuticle bars of *P. tesselatum* and *P. longispiculum* sp. nov. Eventually these additional characteristics of the precloacal supplements may prove to be important to species delimitation.

### Pore complex and lateral pore-like structures

The numbers of longitudinal rows of pore complexes present on the cuticle and the orientation of the slit-like aperture are features already used to differentiate between the genera *Longicyatholaimus* and *Marylynnia* (Hopper, 1972). However, these characteristics are not constant within other genera of the family. *Biarmifer* species, for example, have four or eight longitudinal rows of these structures, and the apertures are oriented transversely, longitudinally, or diagonally. The same is true for *Paracanthonchus* Micoletzky, 1924, where the common pattern is the presence of eight longitudinal rows, but with variations among species. For example, *P. platti* Vadhyar, 1980 has six rows, while *P. kamui* Kito, 1981 and *P. perspicuus* Kito, 1981 have twelve. The pore complex structure is rarely cited in *Pomponema* species descriptions, but for those that have been described, they appear as four sublateral longitudinal rows (Lorenzen, 1972; Ott, 1972; Blome, 1974; Gourbault, 1980), and the pore aperture may be longitudinally or diagonally oriented (Hopper, 1972; present study).

Lateral pore-like was analyzed in detail in only a few species of Cyatholaimidae, and their overall structure is similar among them: it is formed by a cuticularized open, supported by punctations fused or not, and with or without a central dome (present study; Leduc & Zhao, 2016). The variation is in the number and size of LPs throughout the body and in the number of punctations that supported it. The pattern that seems to be more common among the Cyatholaimidae species is the opening supported by unmodified punctations (not fused), as observed in *Biarmifer nesiotes* sp. nov., *L. cervoides* Vitiello, 1970, *L. complexus* Warwick, 1970, *Marylynnia bellula* Vitiello, 1970 Hopper, 1977, *Metacyatholaimus adriaticus* Vidakovic, Travizi & Boucher, 2003 and *M. chabaudi* Gourbault, 1980. In these species, there is one punctation supporting each of the anterior and posterior extremities of the opening rings. However, in *M. bellula* and *L. cervoides* there are a few LPs throughout the body with three or four punctations supporting the ring. Different from all other species so far, in *Pomponema longispiculum* sp. nov. the opening is supported by two to six unmodified punctations. The location of LPs between the mediolateral and laterodorsal lines in the mid-body region of *Pomponema longispiculum* sp. nov. is related to the beginning of the lateral differentiation of longitudinal rows of punctations. Since those structures are rarely cited in species descriptions, it is not clear how usual this pattern of LPs distribution is within the family.

A second pattern was described for a few species, where the cuticularized ring is supported by modified (broad and curved) or possibly fused punctations. In *Metacyatholaimus delicatus* Leduc & Zhao, 2016 and *Paracanthonchus miltommatus* Leduc & Zhao, 2016, the opening of the LP is supported by two modified or fused punctations. The LP is not described in full detail in the description of *P. mamubiae* Miljutin & Miljutina, 2015, but based on their SEM photographs, the opening also seems to be supported by modified punctations.

## Conclusions

The present study contributes to the growing knowledge of the diversity of free-living marine nematodes from the Brazilian coast. One of the new species described is classified in *Biarmifer*, a genus with few registers globally, and recorded for the first time in the country. The second is a *Pomponema* species, a genus very speciose and commonly found on surveys on the coast of Brazil. The taxonomy of the *Biarmifer* genus will be beneficiated from a detailed description and comparison of the structure of the spicules among species. For *Pomponema*, the characterization of the precloacal supplements in the ventral view may reveal previously overlooked features that may be taxonomically informative. The structures present in the cuticle can be useful to differentiate among some genera and species of the family. The organization of pore complex in longitudinal rows may be more relevant to genus identification and the number of those structures by row seems to be useful in species delimitation. Nevertheless, there is still not enough information about these cuticle structures across the family to endorse their overall phylogenetic importance and detailed morphological data acquisition may help to clarify this issue.

##  Supplemental Information

10.7717/peerj.14712/supp-1 Table S1 Tabular key of *Biarmifer* species When different from males, the information about females is provided between brackets.

10.7717/peerj.14712/supp-2 Table S2 Tabular key of *Pomponema* species When different from males, the information about females is provided between brackets. Types of lateral differentiation of cuticle (L.d.): (1) Two to four longitudinal rows of dots not connected transversely, (2) Two to four longitudinal rows of dots connected transversely by lines, (3) Widely spaced dots, (4) T wo longitudinal rows of bigger dots with wide space between them.

## Additional Information and Declarations

### Field Study Permissions

The following information was supplied relating to field study approvals (*i.e.*, approving body and any reference numbers): Collection was approved by the ICMBio (number: 19887-1).

### DNA Deposition

The following information was supplied regarding the deposition of DNA sequences: The rDNA SSU sequence is available at GenBank: OP548510.

### Data Availability

The following information was supplied regarding data availability: The raw data is available in Table 1 and in the Figures. The specimens are reposited in the MDBio Nematode Collection (Instituto de Biologia—Unicamp, Cidade Universitária Zeferino Vaz, Rua Charles Darwin, s/n—Bloco N,13.083-863, Barão Geraldo–Campinas–SP–Brasil): *Biarmifer nesiotes* sp. nov.: Holotype male (ZUEC-NMA 35), paratypes (ZUEC-NMA 36-43). *Pomponema longispiculum* sp. nov.: Holotype male (ZUEC-NMA 50), paratypes (ZUEC-NMA 51-58).

### New Species Registration

The following information was supplied regarding the registration of a newly described species: Publication LSID: urn:lsid:zoobank.org:pub:2430E61E-EDC6-410B-B7CE-B24C1AA6CA0B. Biarmifer nesiotes LSID: urn:lsid:zoobank.org:act:F5253EA9-84EA-4409-B033-34F53DDDCEE4. Pomponema longispiculum LSID: urn:lsid:zoobank.org:act:AE58C8EA-4EF9-4ED7-9265-AAA07EFEBCBA.
