# Supplementary material for: Two new species of Cyatholaimidae (Nematoda: Chromadorida) from the Southeastern Brazilian coast with emphasis on the pore complex and lateral pore-like structures
Source: PeerJ. 2023 Feb 20;11:e14712. doi: 10.7717/peerj.14712 (PMC9948750; doi:10.7717/peerj.14712)
Supplement: Table S1 — When different from males, the information about females is provided between brackets. [file peerj-11-14712-s001.docx]

**Table S1: Tabular key of *Biarmifer* species.** When different from males, the information about females is provided between brackets.

| Species | Lateral differentiation of cuticle | P.c. | a | b | c | c' | Amphideal turns | Amphid cdb % | Spicule length (µm) | Nº Supplem. |
| --- | --- | --- | --- | --- | --- | --- | --- | --- | --- | --- |
| *B. cochleatus* Wieser, 1954 | A | 4 | 20-28 | 5-7.5 | 6.1-9 | 4.5-5 | 4.5 | 37 | 60 | 5 |
| *B. dayi* (Inglis, 1963) Cunha *et al*., 2022 | A | 4 | 31 | 4.8 | 12.4 | 3 | 3.75 | 20 | 65 | 0 |
| *B. hopperi* (Sharma & Vincx, 1982) Cunha *et al.*, 2022 | A | 8 | 25.1-36.5 (18-25.5) | 6.8-7.8 | 9.9-10.2 (8.8-9.6) | 3.3-5.2 (6.7) | 5 | 45 | 88 | 5 |
| *B. laminatus* Wieser, 1954 | P^1^ | 4 | 40 (20.5 -25.9) | 8 (5.2-6.1) | 12 (11.4-15.6) | 3.6 (2.75-3) | 8.25 (5.5) | 54 (50) | 32 | n.a |
| *B. madrynensis* Pastor de Ward, 2001 | P | 4 | 27.2-45.3 | 4.7-6.3 | 5.3-8 | 8-9 | 3.5 | 20-32.5 | 62-78 | 3 |
| *B. nesiotes* sp. nov. | P | 8 | 17.4-26.6 | 5.8-6.8 | 7.3-10.5 | 3.5-4.2 | 5 (4-5) | 47-50 (40-46) | 72-84 | 5 |
| *B. punctata* (Jensen, 1985) Cunha *et al*., 2022 | P | 4 | 29-30 | 6.2 (4.9) | 8.4 (6.6) | 5 (6.5) | 5 | 53 | 60 | 6 |

^1^Lateral differentiation was observed only on the tail.

Abbreviations: a, body length/maximum body diameter; b, body length/pharynx length; c, body length/tail length; c′, tail length/anal or cloacal body diameter; n.a, information not available; P.c., number of longitudinal rows of pore-complex. A, absent; P, present.

**REFERENCES**

Cunha BP, Fonseca G, Amaral ACZ. 2022. Diversity and distribution of Cyatholaimidae (Chromadorida: Nematoda): a taxonomic and systematic review of the world records. Frontiers in Marine Science 9:836670. DOI 10.3389/fmars.2022.836670.

Inglis WG. 1963. New marine nematodes from off the coast of South Africa. Bulletin of British Museum Natural History (Zool.) 10(9):529–552.

Jensen P. 1985. The nematode fauna in the sulphide-rich brine seep and adjacent bottoms of the East Flower Garden, NW Gulf of Mexico. 1. Chromadorida. Zoologica Scripta 14:247–263. DOI 10.1111/j.1463-6409.1985.tb00195.x.

Pastor de Ward CT. 2001. A new nematode from West Patagonian coasts, *Biarmifer madrynensis* sp. n. with a redefinition of the genus *Biarmifer* Wieser, 1954 (Nematoda, Cyatholaimidae). Biologie 71:139–149.

Sharma J, Vincx M. 1982. Cyatholaimidae (Nematoda) from the Canadian Pacific coast. Canadian Journal of Zoology 60:271–280. DOI 10.1139/z82-036.

Wieser W. 1954. Free-living marine nematodes II. Chromadoroidea. Acta Universitatis Lundensis (N.F.2) 50(16):1–148.
