# Supplementary material for: Two new species of Cyatholaimidae (Nematoda: Chromadorida) from the Southeastern Brazilian coast with emphasis on the pore complex and lateral pore-like structures
Source: PeerJ. 2023 Feb 20;11:e14712. doi: 10.7717/peerj.14712 (PMC9948750; doi:10.7717/peerj.14712)
Supplement: Table S2 — When different from males, the information about females is provided between brackets. Types of lateral differentiation of cuticle (L.d.): (1) Two to four longitudinal rows of dots not connected transversely, (2) Two to four longitudinal rows of dots connected transversely by lines, (3) Widely spaced dots, (4) T wo longitudinal rows of bigger dots with wide space between them. [file peerj-11-14712-s002.docx]

**Table S2: Tabular key of *Pomponema* species.** When different from males, the information about females is provided between brackets. Types of lateral differentiation of cuticle (L.d.): (1) Two to four longitudinal rows of dots not connected transversely, (2) Two to four longitudinal rows of dots connected transversely by lines, (3) Widely spaced dots, (4) Two longitudinal rows of bigger dots with wide space between them.

| Species | L.d. | V.t. | R.d. | a | b | c | c' | Amphid. width (%) | Amphid. turns | O.l.s (µm) | Nº Supplem. | Spicule length (µm) |
| --- | --- | --- | --- | --- | --- | --- | --- | --- | --- | --- | --- | --- |
| *P. ammophilum* Lorenzen, 1972 | A | P | P | 28-40 | 5.5-6.8 | 7.7-9 | n.a | ~55 | 5(4.5) | 15-16 | 19-22 | 54-65 |
| *P. astrodes* Lorenzen, 1972 | 1 | P | A | 40-53 | 7 | 4.4-5.6 | n.a | ~51 | 6 | 6 | 11 | 33-35 |
| *P. clavicaudatum* (Schuurmans Stekhoven, 1935) Lorenzen, 1972 | 1 | A | A | 48.1 (36) | 7.3 (5.6) | 8.7 (7.7) | 4.5 | 50 | 5 | 14 (9) | 22 | 48 |
| *P. compactum* Lorenzen, 1972 | 3 | P | P | 36-41 (30-31) | 4.7-5.3 (4.2-4.5) | 7.5-9.2 (5.9-6.7) | n.a | 56-60 (46-48) | 5-5.25 (4-4.5) | 11-16 | 13-16 | 30-35 |
| *P. concinnum* (Wieser, 1954) Lorenzen, 1972 | 2 | A | A | 28 | 5.6 | 7 | 6 | 40 | 4 | 6 | 20 | 36 |
| *P. coomansi* Vincx, 1981 | 2 | P | A | 42.7-47.5 | 6 | 4.7-6.2 | 11 | 38 | 5.3 | 16 | 2 | n.a |
| *P. corniculata* Gourbault, 1980 | 4 | P | A | 22.9-23.7 (20.9-22.4) | 4.3-4.8 | 11.2-13.5 | 2.3-2.5 (3-3.4) | 45 | 6 | 2.5-3 | 20 | 41-47 |
| *P. cotylophorum* (Steiner, 1916) Lorenzen, 1972 | 4 | A | A | 30 | 5 | 12 | n.a | n.a | n.a | A? | 15 | n.a |
| *P. debile* Lorenzen, 1972 | 2 | P | P | 40-45 | 5.5-6.1 | 8.7-9.5 | n.a | 42-46 | 4-5 | 8.5-9.5 | 15-18 | 27-29 |
| *P. elegans* Lorenzen, 1972 | 2 | P | P | 49-60 (41) | 6.5-7.8 | 7.7-9.2 (5.4) | n.a | ~70 (40) | 6 | 8-9 | 12-15 | 30-34 |
| *P. foeticolum* (Ott, 1972) Cidreira *et al*., 2019 | A | P | A | 42.9-60.9 | 7-9.8 | 12.5-18.3 | 3.1-3.8 (4.9) | 44-46 (38) | 5 (3.5) | 11-14 | 13-16 | 39-44 |
| *P. golikovi* Platonova, 1988 | A | P | A | 34.5 | 6.8 | 9.7 | 3.5 | 50 | 5 | 6.5 | 13 | 50 |
| *P. hastatum* (Ott, 1972) Cidreira *et al*., 2019 | 4 | P | A | 47.7-55.5 | 4.7-5 | 17.9-18.2 (14.5-16.3) | 3.1-3.4 (4.7-4.8) | 53-54 (38) | 3.25-3.75 | 11-13 | 19-20 | 25-28 |
| *P. koesterae* Jensen, 1992 | 4 | A | P | 24 (18-21) | 4.2-6.1 | 7.3-7.6 (5.7-7.9) | 5.6-9 | 55 | 10 | 3-4 | 15-17 | 37-41 |
| *P. lineatum* (Gerlach, 1953) Wieser, 1959 | 1 | P | A | 38 (25) | 5.9 (5.6) | 8.8 (7) | 5 (6-7) | 75 | 2.5 | 8-13 | 13 | 30 |
| *P. litorium* (Cobb, 1920) Lorenzen, 1972 | 2 | P | P | 41.7 | 5.8 | 10 | 5.3 | n.a | n.a | n.a | n.a | n.a |
| *P. longispiculum* sp. nov. | 1 | P | P | 28-39.7 | 5.7-7 | 6-9.6 | 4.7-5.8 (6.1-7.5 | 54.8-59.6 (29.2-32.9) | 5.5-6 (4-5) | 11.5-15 | 19-22 | 72.5-78 |
| *P. loticum* Lorenzen, 1972 | 2 | P | P | 30.5-31 (26) | 5.2-5.6 (4.9) | 7.3-84 | 4.6-5 (5.4) | n.a | n.a | 16 | 19 | 35-35.5 |
| *P. macrospirale* (Ott, 1972) Cidreira *et al*., 2019 | 4 | P | A | 45.1-49.7 | 4.2-4.6 | 15.4-16.1 | 3.4-3.6 | 63-76 | 4.75-5.75 | 15-16 | 18 | 27-30 |
| *P. mirabile* Cobb, 1917 | n.a | P | A? | 52.6 | 4.8 | 13.7 | 4.9 | ~75 | 6 | n.a | 20 | n.a |
| *P. multipapillatum*^1^ (Filipjev, 1922) Wieser, 1954 | 2 | P | P | 18-30 | 5.5-7.6 | 7.3-9.6 | n.a | n.a | 5 | 4-8 | 15-19 | 34-46 |
| *P. polydontus* Murphy, 1963 | 1 | P | P | 43.1-63.2 | 5.4-7.4 | 9.9-12.5 | 6 (5.2) | 35 | 4.5 | 23 | 24 | 55 |
| *P. proximamphidum* Tchesunov, 2008 | 4 | P | P | 27.6-34 | 4.7-6.2 | 4-6 | 7.4-8.1 | 55-59 | 5 | 2-5.5 | 13-14 | 41-46 |
| *P. reductum* Warwick, 1970 | 1 | P | P | 43.4-46.2 (33.3-42.6) | 6.2-7 | 9.7-10.9 | 4.5-5.5 (5.6-6) | 37-42 (27-31) | 4-4.5 (3.5) | 8-10 | 20-24 | 43-46 |
| *P. sedecima* Platt, 1973 | 2 | P | P | 48.1-49.5 (37.5) | 7-7.4 (6.5) | 12.2-12.9 (10.4) | 3.9-4.9 | 32-35 | 3.5 | 9-10 | 15-17 | ~32 |
| *P. segregatum* Wieser, 1959 | 4 | P | A | 24 | 6 | 9.1 | 4.8 | 48 | 4 | 14 | 9 | 26 |
| *P. stomachor* Wieser, 1954 | 1 | P | P | 35-40.7 (31.6-34.1) | 6.2-6.6 (6.5-8.1) | 7.1-8 (6.4-7.3) | 7 | 50 | 6-6.5 (4-5) | 18 (9) | 23 | 75 |
| *P. syltense* Blome, 1974 | A | P | P | 21-24 (17-19) | 4.7-5.2 | 4.8-6.5 | 4.5-6.5 | 37-43 (28-29) | 3.5 | 24 | 26-29 | 45-50 |
| *P. tautraense* (Allgén, 1933) Lorenzen, 1972 | A | A | A | 46.6 | 7.3 | 8.3 | n.a | ~50 | 4.5 | n.a | 12 | n.a |
| *P. tesselatum* Wieser & Hopper, 1967 | 1 | P | A | 36.4 | 4.25 | 11.6 | 3.6 | 68 | 4.5 | 16 | 15 | 45 |
| *P. veronicae* Cidreira *et al*., 2019 | 2 | P | P | 38.9-45.9 (31.6-35.8) | 5.9-6.2 | 6.9-7.4 (5.7-7.2) | 6.8-7.9 (8.9-9.7) | 44-50 (33-34) | 4.5 (3.5) | 20-23 (18-19) | 20 | 44-47 |
| *P. websteri* (Sharma & Vincx, 1982) Cidreira *et al.* 2019 | A | P | A | 80.5-88.4 (64.4) | 13.4-14.9 | 7.1-10.1 (5.7) | 12 (18) | 58 (41) | 5.5 | 4 | 13-15 | 42 |

^1^Data from Lorenzen (1972)

Abbreviations: a, body length/maximum body diameter; b, body length/pharynx length; c, body length/tail length; c′, tail length/anal or cloacal body diameter; cbd, corresponding body diameter; n.a, information not available; O.l.s: outer labial setae length; V.t.: ventral teeth; R.d.: rows of denticles on the buccal cavity. A: absent; P: present.

**REFERENCES**

Allgén, C.A. 1933. Freilebende Nematoden aus dem Trondhjemsfjord. Capita Zoologica 4 (2):1–162.

Blome, D. 1974. Zur systematik von Nematoden aus dem Sandstrand der Nordseeinsel Sylt. Mikrofauna Meeresbodens 33:77–99.

Cidreira, G., Pinheiro-Junior, E.P., Venekey, V. & Alves, O.F.S. 2019. A new species of *Pomponema* Cobb, 1917 (Nematoda: Cyatholaimidae) from Northeast of Brazil, with reference to the taxonomic status of the genera *Parapomponema* Ott, 1972 and *Propomponema* Ott, 1972. Zootaxa 4691:63–77. DOI 10.11646/zootaxa.4691.1.5

Cobb, N. A. 1917. Notes on Nemas. Contributions to a Science of Nematology 5:117–128.

Cobb, N.A. 1920. One hundred new nemas (type species of 100 new genera). Contributions to Science of Nematology 9:217–343.

Filipjev, I.N. 1922. Encore sur les Nématodes libres de la mer Noire. Tr. Stravrop. Skh. Inst. Zool. 1:83–184.

Gerlach, S.A. 1953. Die Nematodenbesiedlung des Sandstrandes und des Küstengrundwassers an der italienischen Küste I. Systematischer Teil. Archivio Zoologico Italiano 37:517–640.

Gourbalt, N. 1980. Nématodes abyssaux (Campagne Walda du N/O "Jean Charcot"). I. Espèces nouvelles de Cyatholaimidae. Cahiers de Biologie Marine 21:61–71.

Jensen P. 1992. Predatory nematodes from the deep-sea: description of species from the Norwegian Sea, diversity of feeding types and geographical distribution. Cahiers de Biologie Marine 33:1–23.

Lorenzen, S. 1972. Die Nematodenfauna im Verklappungsgebiet für Industrieabwässer nordwestlich von Helgoland: III. Cyatholaimidae, mit einer Revision von *Pomponema* Cobb, 1917. Veröffentlichungen des Instituts für Meeresforschungen in Bremerhaven 13:285–306.

Murphy, D.G. 1963. A new genus and two new species of nematodes from Newport, Oregon. Proceedings of the Helminthological Society of Washington 30:73–78.

Ott, J.A. 1972. Twelve new species of nematodes from an intertidal sandflat in North Carolina. Internationale Revue der gesamten Hydrobiologie und Hydrographie 57:463–496.

Platonova, T.A. 1988. New species of free-living Nematodes from the White Sea *Pomponema golikovi* sp. n. (Nematoda. Chromadorida). Issledovaniya Fauny Morei 3846:88–90.

Platt, H.M. 1973. Freeliving marine nematodes from Strangford Lough, Northern Ireland. Cahiers de Biologie Marine 14:295–321.

Schuurmans Stekhoven, J.H.Jr. 1935. Freeliving Marine Nemas of the Belgian Coast. I. and II. Mémoires du Musée royal d'histoire naturelle de Belgique 72:1–36.

Sharma, J. & Vincx, M. 1982. Cyatholaimidae (Nematoda) from the Canadian Pacific coast. Canadian Journal of Zoology 60:271–280.

Steiner, G. 1916. Freilebende Nematoden aus der Barentssee. Zoologische Jahrbücher 39:511–664.

Tchesunov, A.V. 2008. Three new species of free-living nematodes from the South-East Atlantic Abyss (DIVA I Expedition). Zootaxa 1866:151–174.

Vincx, M. 1981. New and little known nematodes from the North sea. Cahiers de Biologie Marine 22:431–451.

Warwick, R.M. 1970. Fourteen new species of free-living marine nematodes from the Exe estuary. Bull. Br. Mus. nat. Hist. (Zool.) 19 (4):137–177.

Wieser, W. 1954. Free-living marine nematodes II. Chromadoroidea. Acta Universitatis Lundensis (N.F.2) 50 (16):1–148.

Wieser, W. 1959. Free-living nematodes and other small invertebrates of Puget Sound beaches. University of Washington Publications in Biology (University of Washington Press, Seattle) 19:1–179.

Wieser, W. & Hopper, B. 1967. Marine Nematodes of the East Coast of North America. I. Florida. Bulletin Museum of Comparative Zoology 135(5):239–344.
